# Assessment of the biological efficacy of gold *Nannochloropsis oculata* nano-extract against lithium-induced toxicity in rats

**DOI:** 10.1038/s41598-025-33835-5

**Published:** 2026-01-26

**Authors:** Randa Abdel-Salam Bekheit, Wael Mahmoud Aboulthana, Waleed Mohamed Serag

**Affiliations:** 1 Chemistry Department (Biochemistry Branch), Faculty of Science, Suez University, P.O. 43518, Suez, Egypt; 2 Biochemistry Department, Biotechnology Research Institute, National Research Centre, 33 El Bohouth St., P.O. 12622, Dokki, Giza Egypt

**Keywords:** Lithium toxicity, *Nannochloropsis oculata*, Gold nanoparticles, Green nanotechnology, Electrophoresis, Gene expression, Biochemistry, Biological techniques, Biotechnology, Physiology

## Abstract

Chronic renal impairment can be caused by prolonged exposure to lithium (Li), which is considered a prototype medication for the treatment of bipolar disorders (BDs). The current study aimed to enhance the biological efficacy of biosynthesized gold nanoparticles (Au-NPs) using an algal extract from *Nannochloropsis oculata* to mitigate the neurotoxicity and kidney damage induced by Li in rats. The kidney and brain tissues were analyzed using conventional biochemical tests to assess oxidative stress and inflammatory responses. Electrophoretic techniques were used to examine native protein and isoenzyme patterns. Histopathological analysis was conducted on both tissues. mRNA expression levels of inflammatory markers (NF-κB, IL-6, and IL-1β) and antioxidant enzymes (SOD1, CAT, and GPx1) were also measured. The study found that the gold *N. oculata* nano-extract restored normal hematological and biochemical parameters that were affected by an injection of lithium carbonate (Li_2_CO_3_). It improved antioxidant indicators, reduced inflammatory markers, and enhanced the antioxidant state of kidney and brain tissues. The gold nano-extract also reduced histopathological damage to the brain and kidney caused by Li_2_CO_3_. Furthermore, it alleviated the differences in electrophoretic patterns between the Li_2_CO_3_-injected group and the control group. The nano-extract also restored mRNA expression of inflammatory markers and antioxidant enzymes that were altered by Li_2_CO_3_ intoxication. The study found that the gold *N. oculata* nano-extract reduced kidney and brain toxicity at various levels, including biochemical, histopathological, physiological, and molecular.

**Supplementary Information:**

The online version contains supplementary material available at 10.1038/s41598-025-33835-5.

## Introduction

Bipolar disorders (BDs) are severe affective disorders characterized by alternating (hypo)manic, depressive, and mixed phases, with periods of euthymia in between^1^. Treatment for BDs often involves lifelong management. Valproate and carbamazepine, commonly used anti-convulsants, help alleviate depressive symptoms in BDs^2^ but may also induce a switch to hypomania or mania. Therefore, they are often combined with lithium (Li), which is the gold standard for preventing recurrences in BDs and effectively treats mania and maintains bipolar depression^3^.

Lithium salts are the standard treatment for bipolar disorders due to their effectiveness in treating acute phases, particularly mania/hypomania, and reducing the risk of relapse in both mood polarities^4^. Predictors of a positive response to Li include a family history of BDs and positive response to Li , consistent intervals between mood episodes, classic cycles of mania, depression, and intervals, absence of comorbidities, absence of mixed features, and a lower number of episodes before starting Li treatment^5^. Abrupt discontinuation of Li can lead to a higher risk of recurrence compared to before starting treatment, which is considered an iatrogenic harm known as the rebound phenomenon^6^.

Prolonged exposure to Li during BDs therapy can result in chronic tubulointerstitial nephritis, leading to chronic kidney disease (lithium nephropathy), hyperparathyroidism, and hypercalcemia^7^. Therefore, close monitoring of these aspects is essential to minimize the risk of endocrinological and renal side effects from Li . Recent research has focused on identifying natural antioxidants that can be combined with anti-epileptic drugs to protect renal tissues from oxidative damage caused by free radicals^8,9^.

Marine organisms are rich sources of biologically active metabolites, some of which are being developed into new drugs^10,11^. Algae, in particular, have garnered global attention for their production of valuable natural products^12^. Extracts from *Nannochloropsis oculata* have demonstrated antioxidant, antibacterial, anti-fungal, anti-inflammatory, and anti-cancer properties^13^. The active compounds in *N. oculata* extract reduce free radical production and boost antioxidant activity, due to its high protein, polyunsaturated fatty acids, and antioxidant pigment content^14,15^.

Biologically active components with large molecular weights have slow absorption rates, limiting their ability to penetrate cell membranes and reducing their bioavailability and potency^16^. Incorporating nanotechnology can enhance the efficacy of natural products by reducing toxicity and improving bioavailability, allowing for less frequent dosing and lower toxicity^17^. Nanoparticles can boost the activity and effectiveness of plant extracts, reducing the need for frequent administration and decreasing toxicity^18^. Nano-extracts, created by incorporating metal nanoparticles (M-NPs) into plant extracts, improve stability^19,20^. Biosynthesized M-NPs using natural extracts enhance biological activity at lower concentrations compared to plant extract alone, increasing practical uses^21,22^. Phytochemicals in marine algae, such as hydroxyl, carboxyl, and amino functional groups, can act as metal-reducing and capping agents, resulting in a robust coating on gold nanoparticles (Au-NPs) with impressive antioxidant, radical scavenging, anti-bacterial, and cytotoxic properties^23^.

Recent research has demonstrated that biofabricated Au-NPs have potent scavenging activity against free radicals by transferring electrons or hydrogen. The increased surface area of Au-NPs is believed to enhance their effectiveness^24^. These nanoparticles exhibit anti-diabetic properties by inhibiting carbohydrate-metabolizing enzymes (α-amylase and α-glucosidase), which hinders starch breakdown^25^. Additionally, the strong affinity of acetylcholinesterase (AChE) enzyme for Au-NPs can alter its structure and inhibit AChE activity, contributing to the anti-Alzheimer effects of Au-NPs^26^. Biosynthesized Au-NPs can stabilize tertiary and quaternary protein structures, preventing protein denaturation and exhibiting anti-inflammatory properties^27^.

Biosynthesized Au-NPs have demonstrated cytotoxic activity against various cancer cells by regulating apoptosis, altering the expression of anti-apoptotic genes, and interfering with signal transduction pathways to increase reactive oxygen species (ROS) accumulation, ultimately promoting cellular death^28,29^.

The study aimed to improve the biological efficiency of Au-NPs produced using *N. oculata* algal extract in reducing lithium-induced toxicity in rat kidney and brain tissues. This was evaluated through biochemical, histological, physiological, and molecular assessments.

## Materials and methods

### Administration of the algal extract

Bekheit et al.^30^ determined the median lethal doses (LD_50_) of native *N. oculata* algal extract and gold nano-extract to be approximately 5000.00 and 9333.33 mg/kg, respectively. Therapeutic dosages of 250 and 466.67 mg/kg b.w. were selected for oral administration in the in vivo study, calculated as 1/20 of the LD_50_ values.

### Induction of toxicity

Rats were given a solution of Li_2_CO_3_ in saline intraperitoneally (*i.p.*) at a dose of 25 mg/kg body weight twice daily for 30 days, as shown by Leschiner et al.^31^.

### Experimental design

Sixty (60) adult male Wistar rats (120-150 g) were maintained in the Animal House under controlled environmental conditions (25 ± 2 °C) at the National Research Centre in Giza, Egypt. The study was approved by the Ethical Committee of the Faculty of Science, Suez University, Suez, Egypt. All methods were conducted in compliance with the appropriate guidelines and regulations. The current study is reported following ARRIVE guidelines. The rats were fed a standard diet and given normal tap water, and housed in six cages with ten rats per cage.

Control group: Rats received distilled water for 28 days. *N. oculata* algal extract treated group: Rats were treated with *N. oculata* extract orally at a dose of 250 mg/kg for 21 days. Gold *N. oculata* algal nano-extract treated group: Rats were treated with gold nano-extract orally at a dose of 466.67 mg/kg for 21 days. Li_2_CO_3_ intoxicated group: Rats were given Li_2_CO_3_ for 30 days. Li_2_CO_3_ + *N. oculata* algal extract treated group: Rats were given Li_2_CO_3_ for 30 days and treated at the same time with native algal extract for 21 days. Li_2_CO_3_ + Gold nano-extract treated group: Rats in this group were given Li_2_CO_3_ for 30 days and treated at the same time with gold nano-extract for 21 days.

### Collection of blood samples and preparation of tissues

After the final treatment dose, the rats underwent an 18-h fast and were anesthetized with xylazine and ketamine (20 and 50 mg/kg, intraperitoneally). Blood samples were collected from the retro-orbital plexus and placed in heparinized tubes for hematological analysis. The remaining blood samples were centrifuged at 3000 rpm for 15 min after clotting, and the separated sera were kept at − 20 °C until the biochemical analysis. All rats were euthanized by cervical dislocation under deep anesthesia. Kidney and brain tissues were then extracted from the animals and rinsed in ice-cold saline. A few autopsied fragments were kept for histological analysis in neutral buffered formalin solution (10%), while the remaining fraction was homogenized in potassium phosphate buffer (pH 7.4) and centrifuged for 10 min at 3000 rpm. The clear supernatants were stored at − 80 °C for use in biochemical assays. The remaining tissues were quickly frozen with liquid nitrogen for molecular and electrophoretic assays.

### Hematological and biochemical assays

Hematological measures, including red blood cell (RBC) and white blood cell (WBC) counts, as well as differential blood cell counts, were conducted on heparinized blood samples using an automated blood analyzer. Traditional biochemical measurements for liver, heart, and kidney functions, along with lipid profiles (total cholesterol (TC), triglycerides (TG), and high-density lipoprotein-cholesterol (HDL-C)), were carried out in serum specimens using commercially available kits from Spectrum Diagnostics Egyptian Company for Biotechnology, Cairo, Egypt. Low-density lipoprotein-cholesterol (LDL-C) levels were calculated using the formula proposed by Schumann and Klauke^32^.

### Biochemical assays in supernatants of tissues homogenates

Oxidative stress markers, including total antioxidant capacity (TAC) and reduced glutathione (GSH), were measured in kidney and brain tissue homogenates following the protocols by Koracevic et al.^33^ and Beutler et al.^34^, respectively. Antioxidant enzyme activities of superoxide dismutase (SOD), catalase (CAT), and glutathione peroxidase (GPx) were quantified per gram of tissue using methods by Sun et al.^35^, Aebi^36^, and Paglia and Valentine^37^, respectively. Lipid peroxidation product (LPO) and total protein carbonyl content (TPC) were assessed in nmol/g tissue and nmol/mg tissue, respectively, based on procedures by Ohkawa et al.^38^ and Levine et al.^39^. Inflammatory markers including tumor necrosis factor-α (TNF-α)^40^ and interleukin-6 (IL-6)^41^ were measured in pg/g using enzyme-linked immunosorbent assay (ELISA). Acetylcholinesterase (AChE) activity was determined as nmol ACh hydrolyzed/min/mg protein following the Ellman Method^42^ and modified by Gorun et al.^43^.

### Histopathological examination

Autopsy samples from rats’ kidneys and brains were preserved in 10% formal saline for 24 h. The samples were washed in tap water, dehydrated with alcohol (methyl, ethyl, and absolute ethyl), and cleaned with xylene before embedding in paraffin at 56 °C for 24 h. Tissue blocks were sectioned at 4 μm thickness using an LEITZ ROTARY microtome. The sections were mounted on glass slides, deparaffinized, and stained with hematoxylin and eosin (H&E) for examination under a light microscope following the method demonstrated by Banchroft et al.^44^. The severity of histopathological lesions was assessed and graded from five microscopic fields per rat using the classification system by Dommels et al.^45^: no changes (0–25%), mild (25–50%), moderate (50–75%), and severe changes (75–100%).

### Electrophoretic assays

#### Native electrophoretic patterns

Equal amounts of kidney and brain tissues (0.2 g each) were homogenized in 1 mL of extraction buffer and centrifuged at 10,000 rpm for 5 min. The supernatants from each group were pooled together to create a single sample. The total protein concentration in the combined samples was determined using the Bradford method^46^. Samples were diluted with loading dye to ensure uniform protein concentrations for electrophoretic assays.

The native proteins separated by Polyacrylamide Gel Electrophoresis (PAGE) were stained with Coomassie Brilliant Blue (CBB)^47^ to visualize protein bands as blue, Sudan Black B (SBB)^48^ to visualize lipid bands as black, and Alizarin Red "S"^49^ to visualize calcium bands as yellow.

The native isoenzymes were separated by electrophoresis and detected using different staining methods. Catalase (CAT) types were identified as yellow bands by incubating the gel with hydrogen peroxide (H_2_O_2_) and staining with Potassium Iodide (KI)^50^. Peroxidase (POX) types were detected as brown bands using Benzidine staining^51^. Esterase (EST) isoenzymes were identified by incubating the gel in a conditioning buffer and staining with a reaction mixture containing Fast Blue RR and α- and β-naphthyl acetate as substrates. α-EST types appeared as brown bands, while β-EST types appeared as pink bands^52^.

#### Data analysis

The native PAGE plates were analyzed with Quantity One software (Version 4.6.2) to determine the relative mobility (Rf), intensity (Int.), and percentage (B%) of stained bands. The similarity index (SI%) and genetic distance (GD%) were calculated using the equation by Nei and Li^53^.

### Molecular assay

The expression of mRNA for antioxidant enzymes (SOD1, CAT, and GPx1) and inflammatory markers (NF-κb, IL-6, and IL-1b) was measured in kidney and brain tissues. The tissue samples were homogenized in TRIzol® Reagent to isolate total RNA (Invitrogen, USA). To ensure the integrity of the extracted RNA, formaldehyde-containing agarose gel electrophoresis was performed, followed by ethidium bromide-stain examination of the 28S and 18S bands. The DNA residues were digested by treating total RNA with RQ1 RNAse-free DNAse (1U) and resuspended in DEPC-treated water. During reverse transcription, the entire Poly(A) + RNA extracted from tissue samples was transformed into cDNA with the RevertAidTM First Strand cDNA Synthesis Kit (MBI Fermentas, Germany). The StepOne™ Real-Time PCR System from Applied Biosystems (Thermo Fisher Scientific, Waltham, MA USA) was used to determine the number of copies in tissue samples. The amplification was performed using *q*Real Time-Polymerase Chain Reaction (*q*RT-PCR) using the following reaction program: 10 min at 95 °C, 15 Sec at 95 °C, and 60 Sec at 60 °C for 40 cycles. The primers with sequences sequences appropriate for the targeted genes are compiled in Supplementary Table 1. The relative mRNA levels were measured using the cycle threshold approach and adjusted to the geometric means of GAPDH, a housekeeping gene^54^. A melting curve analysis at 95.0 °C was done at the end of each *q*PCR to ensure that the primers used are of good quality. Wang et al.^55^ described the 2^*−*ΔΔCT^ method, which was utilized to quantify the targeted genes. All studies on each target gene were performed in triplicate.

### Statistical analysis

Data were analyzed using a one-way analysis of variance (ANOVA) and are presented in Tables and Figures as mean ± standard error (SE). Statistical significance was determined by "*p*" values ≤ 0.05. a: Significant compared to the control group; b: Significant compared to the toxic (Li_2_CO_3_ injected) group.

## Results

### Hematological measurements

Supplementary Table 2 shows that Li_2_CO_3_ intoxication did not induce significant changes in hematological measurements related to red blood cell indices such as RBCs, HB, HCT, MCV, MCH, MCHC, RDW, MPV, and PLT. Compared to the control group, there was a significant (*p* ≤ 0.05) increase in WBC levels and differential counts (Lymph., Mono., Gran.). The levels of WBCs and its differentials dropped significantly (*p* ≤ 0.05) with treatment using *N. oculata* extract compared to the Li_2_CO_3_ injected group, but it was unable to fully restore their levels to normal. The gold nano-extract significantly reduced the abnormal WBC levels (*p* ≤ 0.05), bringing them back to normal values.

### Biochemical measurements

Data from Supplementary Table 3 revealed that the Li_2_CO_3_-injected group exhibited significantly (*p* ≤ 0.05) higher levels of liver enzymes (ALT, AST, ALP, and GGT), kidney function markers (urea, creatinine, and uric acid), heart enzymes (CK, LDH), and lipid profile (TC, TGs, and LDL-c) compared to the control group. Li_2_CO_3_ intoxication led to decreased levels of total protein and albumin. Treatment with *N. oculata* extract improved biochemical parameters but did not fully restore them to normal levels. In contrast, the gold nano-extract completely normalized the biochemical measurements.

### Markers of oxidative stress

Table 1 demonstrates that Li_2_CO_3_ significantly (*p* ≤ 0.05) reduced total antioxidant capacity (TAC) and glutathione (GSH) levels, as well as the activity of antioxidant enzymes (SOD, CAT, and GPx) in kidney and brain tissue homogenates. Treatment with *N. oculata* extract significantly (*p* ≤ 0.05) elevated antioxidant markers compared to the Li_2_CO_3_-treated group, although it did not completely restore normal levels. In contrast, administration of gold nano-extract fully normalized these parameters.

**Table 1:** Effect of gold nanoparticles (Au-NPs) biosynthesized by *N. oculata* algal extract against the changes induced by lithium carbonate (Li_2_CO_3_) in the enzymatic and non-enzymatic markers of the antioxidant system in both kidney and brain tissues of rats.

		C	Algal Ext	Au-Algal Nano-Ext	Li_2_CO_3_	Li_2_CO_3_ treated with	
						Algal Ext	Algal Nano-Ext
Kidney	TAC (µmol/g)	8.87 ± 0.01	8.85 ± 0.02	8.84 ± 0.03	4.41 ± 0.01^a^	6.21 ± 0.01^ab^	8.90 ± 0.02^b^
	GSH (mg/g tissue)	147.64 ± 0.06	145.94 ± 0.06	144.86 ± 0.05	73.82 ± 0.03^a^	98.43 ± 0.04^ab^	146.24 ± 0.06^b^
	SOD (IU/g tissue)	46.58 ± 0.01	45.87 ± 0.02	44.72 ± 0.02	23.29 ± 0.01^a^	31.75 ± 0.02^ab^	45.87 ± 0.02^b^
	CAT (IU/g tissue)	79.59 ± 0.02	80.05 ± 0.02	78.94 ± 0.02	39.94 ± 0.02^a^	55.26 ± 0.02^ab^	80.21 ± 0.03^b^
	GPx(IU/g tissue)	62.54 ± 0.02	63.57 ± 0.02	62.47 ± 0.03	31.27 ± 0.02^a^	45.70 ± 0.02^ab^	61.89 ± 0.02^b^
Brain	TAC (µmol/g)	8.63 ± 0.01	8.55 ± 0.02	8.92 ± 0.01	4.31 ± 0.01^a^	6.17 ± 0.01^ab^	8.76 ± 0.01^b^
	GSH (mg/g tissue)	133.17 ± 0.06	131.86 ± 0.06	132.40 ± 0.07	71.29 ± 0.03^a^	96.72 ± 0.05^ab^	134.15 ± 0.06^b^
	SOD (IU/g tissue)	37.30 ± 0.01	35.39 ± 0.02	37.14 ± 0.02	18.65 ± 0.01^a^	24.20 ± 0.01^ab^	35.48 ± 0.02^b^
	CAT (IU/g tissue)	67.61 ± 0.02	66.84 ± 0.02	65.89 ± 0.02	31.84 ± 0.01^a^	48.79 ± 0.02^ab^	68.08 ± 0.03^b^
	GPx(IU/g tissue**)**	55.82 ± 0.02	57.94 ± 0.02	56.15 ± 0.02	27.41 ± 0.01^a^	40.57 ± 0.01^ab^	55.07 ± 0.02^b^

Data were calculated from five replicates and expressed as mean ± SE.

^a^Significant versus control group, ^b^Significant versus toxic (Li_2_CO_3_) group at *p* ≤ 0.05.

**Fig. 1:**
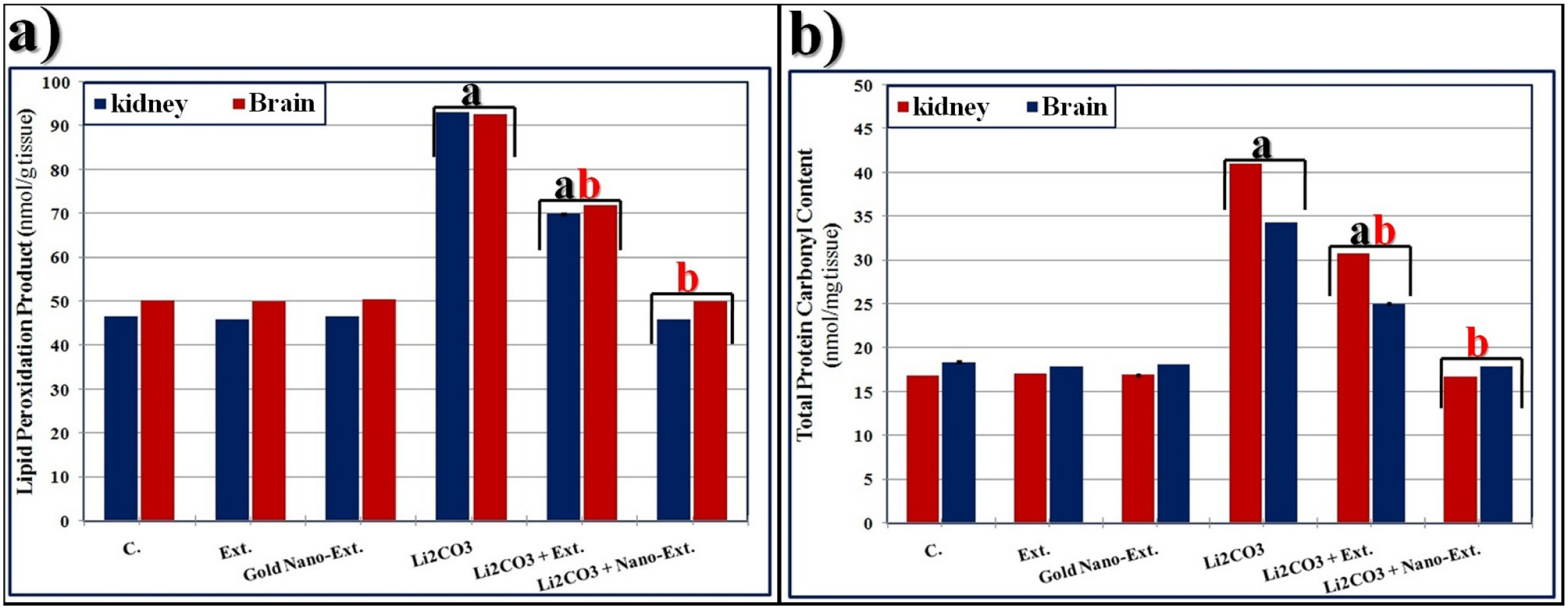
Effect of gold nanoparticles (Au-NPs) biosynthesized by *N. oculata* algal extract against the elevation in (**a**) lipid peroxidation product (LPO), and (**b**) total protein carbonyl content (TPC) induced by lithium carbonate (Li_2_CO_3_) in both kidney and liver tissues of rats. Data were calculated from five replicates and expressed as mean ± SE, (**a**) significant versus control group, (**b**) significant versus toxic (Li_2_CO_3_) group at *p* ≤ 0.05.

Rats exposed to Li_2_CO_3_ showed increased levels of LPO and TPC compared to the control group (Fig. 1). Treatment with *N. oculata* extract significantly (*p* ≤ 0.05) reduced these levels compared to the Li_2_CO_3_ group, but did not fully restore them to normal. In contrast, treatment with gold nano-extract completely normalized the values.

### Inflammatory markers

Levels of TNF-α and IL-6, as well as AChE enzyme activity, are indicators of tissue integrity. Injection of Li_2_CO_3_ led to a significant increase in TNF-α and IL-6 levels (*p* ≤ 0.05) in kidney and brain tissue homogenates. Treatment with *N. oculata* extract resulted in a significant (*p* ≤ 0.05) decrease in these markers compared to the Li_2_CO_3_-intoxicated group, although their levels did not return to normal. Administration of the gold nano-extract significantly (*p* ≤ 0.05) reduced the levels of TNF-α and IL-6 and restored them to normal levels (Table 2).

**Table 2:** Effect of gold nanoparticles (Au-NPs) biosynthesized by *N. oculata* algal extract against the changes induced by lithium carbonate (Li_2_CO_3_) on markers of inflammatory reactions in both kidney and brain tissues of rats.

		C	Algal Ext	Au-Algal Nano-Ext	Li_2_CO_3_	Li_2_CO_3_ treated with	
						Algal Ext	Algal Nano-Ext
Kidney	TNF-α (Pg/g tissue)	276.34 ± 0.45	267.98 ± 0.43	275.00 ± 0.47	552.68 ± 0.87^a^	414.51 ± 0.74^ab^	269.53 ± 0.49^b^
	IL-6 (Pg/g tissue)	335.48 ± 0.69	337.14 ± 0.59	335.07 ± 0.61	670.96 ± 1.37^a^	485.22 ± 1.03^ab^	334.14 ± 0.54^b^
Brain	TNF-α (Pg/g tissue)	114.64 ± 0.71	115.87 ± 0.66	116.50 ± 0.67	229.29 ± 1.44^a^	171.97 ± 1.07^ab^	117.12 ± 0.69^b^
	IL-6 (Pg/g tissue)	137.92 ± 0.27	138.19 ± 0.27	137.15 ± 0.31	275.84 ± 0.63^a^	206.88 ± 0.40^ab^	135.87 ± 0.33^b^

Data were calculated from five replicates and expressed as mean ± SE.

^a^Significant versus control group, ^b^Significant versus toxic (Li_2_CO_3_) group at *p* ≤ 0.05.

**Fig. 2:**
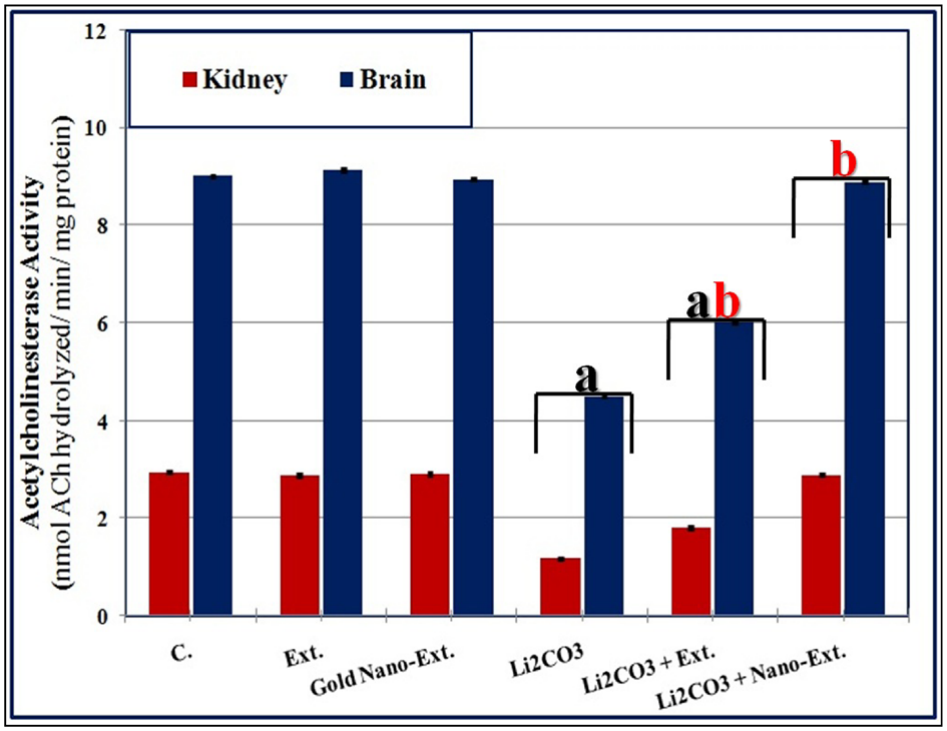
Effect of gold nanoparticles (Au-NPs) biosynthesized by *N. oculata* algal extract against the changes in the activity of acetylcholinesterase (AChE) enzyme induced by lithium carbonate (Li_2_CO_3_) in both kidney and brain tissues of rats. Data were calculated from five replicates and expressed as mean ± SE, (**a**) significant versus control group, (**b**) significant versus toxic (Li_2_CO_3_) group at *p* ≤ 0.05.

Figure 2 shows that Li_2_CO_3_ intoxication significantly (*p* ≤ 0.05) reduced AChE enzyme activity in kidney and brain tissues compared to the control group. The *N. oculata* extract improved inflammatory markers by significantly (*p* ≤ 0.05) increasing enzyme activity compared to the Li_2_CO_3_-intoxicated group, although it did not fully restore activity to normal levels. The gold nano-extract significantly (*p* ≤ 0.05) increased activity and restored it to normal levels in kidney and brain tissues.

### Histopathological examination

**Fig. 3:**
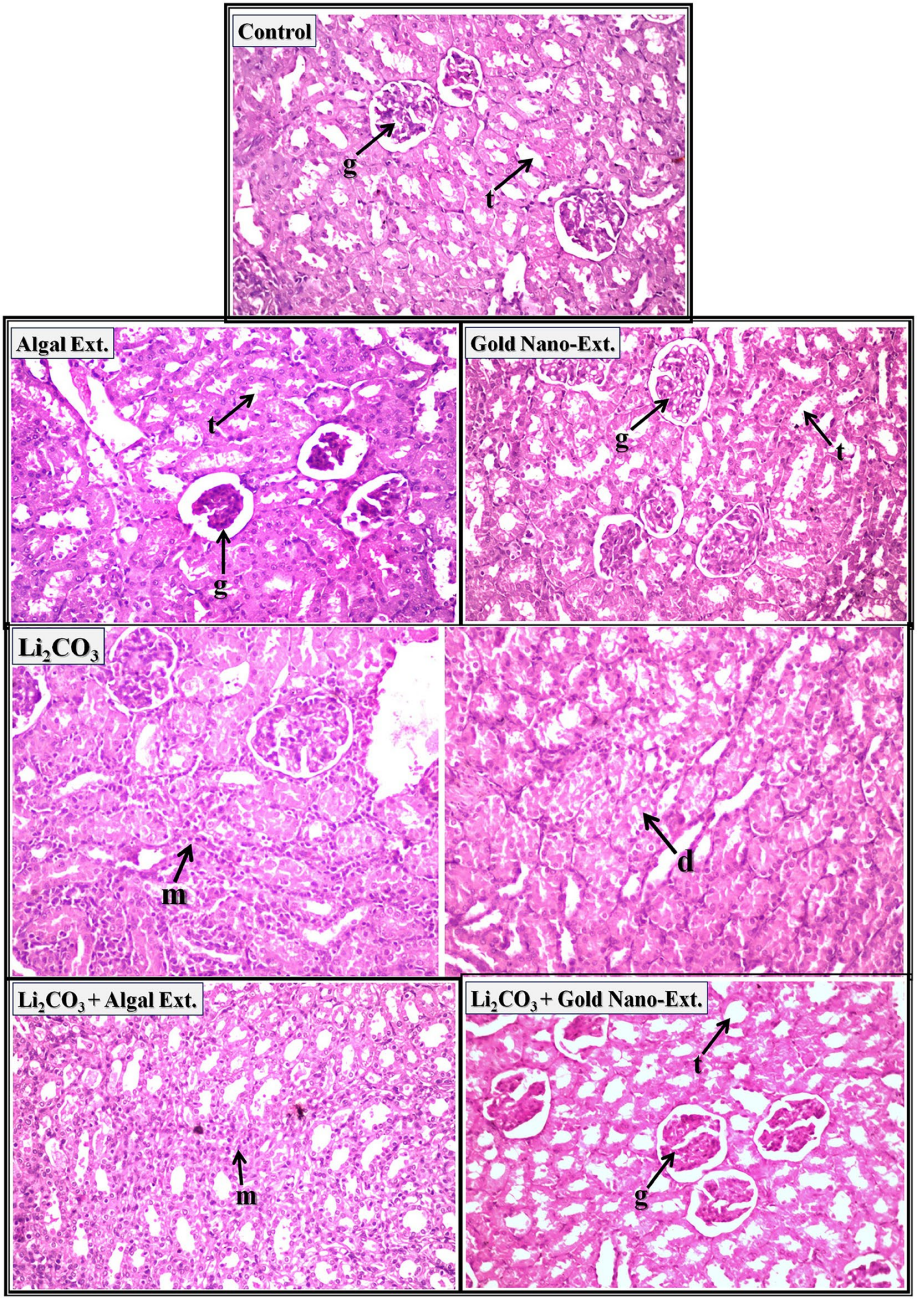
Representative photomicrographs showing the effect of gold nanoparticles (Au-NPs) biosynthesized by *N. oculata* algal extract against the changes induced by lithium carbonate (Li_2_CO_3_) in kidney tissue of rats (H & E-stained, X 40).

In stained kidney sections of control rats, *N. oculata* extract, and gold nano-extract treated rats, light microscopic examination showed normal histological structure of the glomeruli (arrow-g) and tubules (arrow-t) at the cortex (Fig. 3). In contrast, the kidneys of rats intoxicated with Li_2_CO_3_ showed focal inflammatory cells infiltration was detected in between the tubules and glomeruli at the cortex (arrow-m). Moreover, swelling and degeneration were detected in the lining tubular epithelium at the cortex (arrow-d). The examined sections in stained kidney sections of Li_2_CO_3_-intoxicated rats treated with *N. oculata* extract showed focal inflammatory cells infiltration in between the tubules at the corticomedullary portion (arrow-m). In the stained kidney sections of Li_2_CO_3_-intoxicated rats treated with gold nano-extract, normal histological structure of the glomeruli (arrow-g) and tubules (arrow-t) at the cortex were identified and no histopathological alterations were detected compared to the healthy tissue. The histopathological scores in Supplementary Table 4 indicate that the kidneys of rats injected with Li_2_CO_3_ had the most severe damage, with focal inflammatory cell infiltration between tubules (75–100%) and swelling and degeneration of tubular lining epithelium (50–75%). However, these adverse effects were significantly reduced in Li_2_CO_3_-intoxicated rats treated with gold nano-extract (0–25%) compared to those treated with *N. oculata* extract (50–75%).

**Fig. 4:**
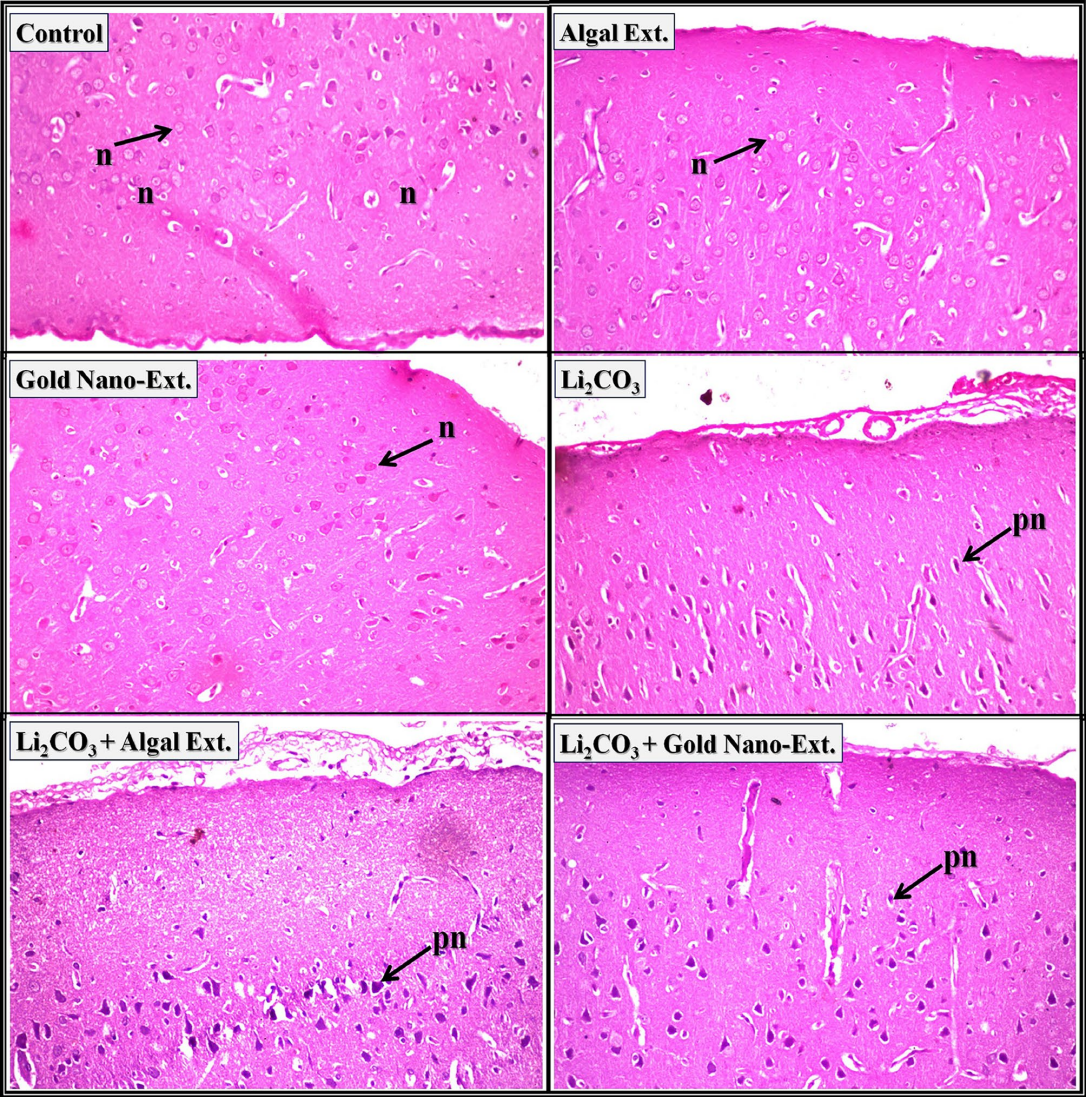
Representative photomicrographs showing the effect of gold nanoparticles (Au-NPs) biosynthesized by *N. oculata* algal extract against the changes induced by lithium carbonate (Li_2_CO_3_) in cerebral cortex in brain tissue of rats (H & E-stained, X 40).

Regarding brain tissue, the cerebral cortex of control rats, *N. oculata* extract, and gold nano-extract treated rats exhibited a normal histological structure of the neurons (arrow-n) (Fig. 4). The cerebral cortex of rats intoxicated with Li_2_CO_3_ revealed nuclear pyknosis and degeneration in the majority of the neurons. In the cerebral cortex of Li_2_CO_3_-intoxicated rats treated with *N. oculata* extract and gold nano-extract, most neurons showed nuclear pyknosis and degeneration (arrow-pn), with no evident amelioration. The histopathological lesion scores in Supplementary Table 5 indicate that the most severe adverse effects (75–100%) were observed in the cerebral cortex of all treated groups.

**Fig. 5:**
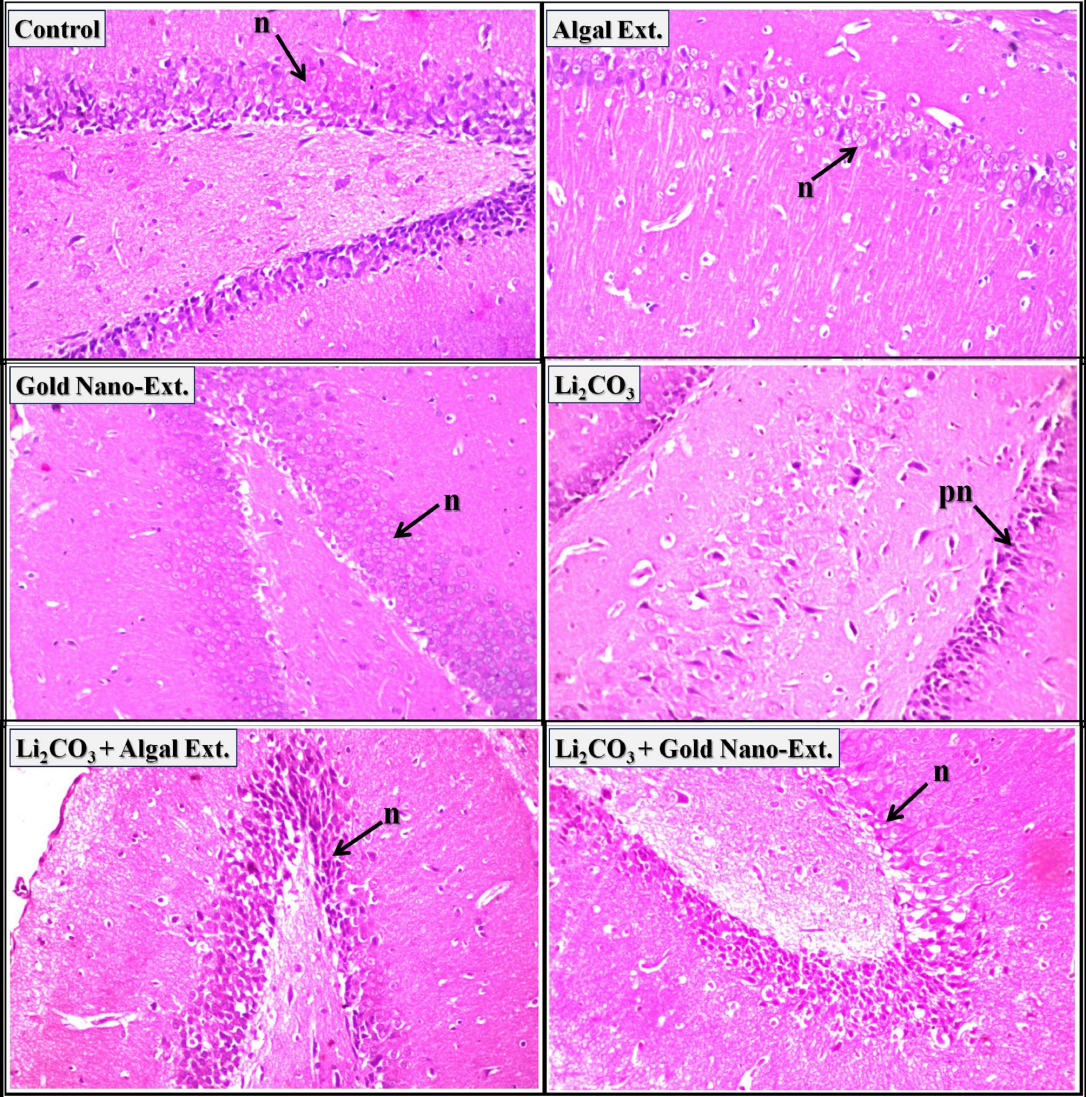
Representative photomicrographs showing the effect of gold nanoparticles (Au-NPs) biosynthesized by *N. oculata* algal extract against the changes induced by lithium carbonate (Li_2_CO_3_) in hippocampus in brain tissue of rats (H & E-stained, X 40).

The normal histological structure of neurons (arrow-n) was observed in the hippocampus of control rats and those treated with *N. oculata* extract and gold nano-extract (Fig. 5). The hippocampus of rats injected with Li_2_CO_3_ revealed nuclear pyknosis and degeneration in some neurons (arrow-pn). Most neurons in the hippocampus of Li_2_CO_3_-intoxicated rats treated with *N. oculata* extract and gold nano-extract showed a normal histological structure (arrow-n). The histopathological scores in Supplementary Table 5 indicate that the hippocampus of rats injected with Li_2_CO_3_ experienced the most severe damage (25–50%). However, these changes were significantly reduced in Li_2_CO_3_-intoxicated rats treated with *N. oculata* extract and gold nano-extract (0–25%).

**Fig. 6:**
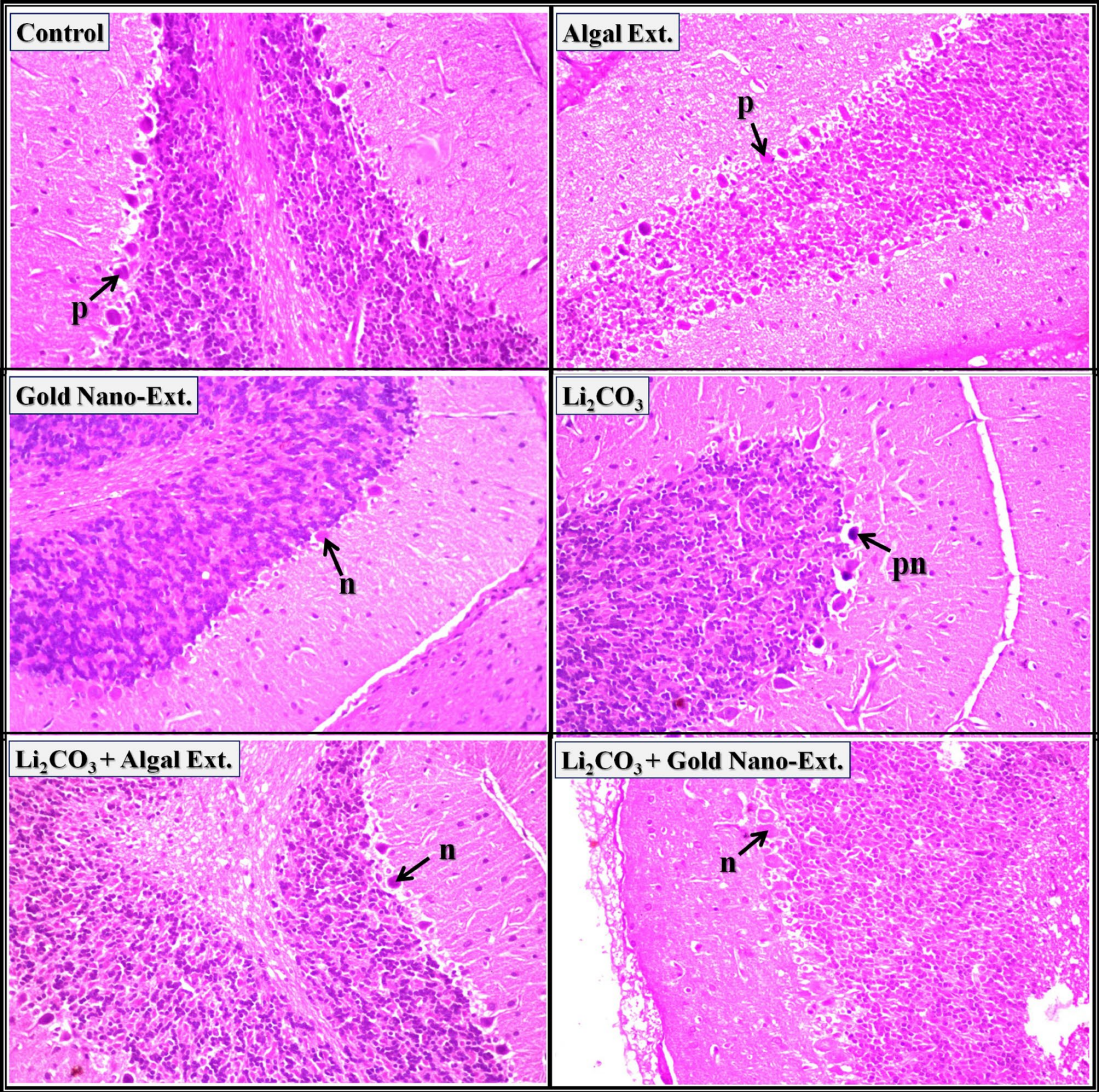
Representative photomicrographs showing the effect of gold nanoparticles (Au-NPs) biosynthesized by *N. oculata* algal extract against the changes induced by lithium carbonate (Li_2_CO_3_) in cerebellum in brain tissue of rats (H & E-stained, X 40).

Purkenji cells with molecular and granular regions (arrow-p) and neurons (arrow-n) had the normal histological structure in the cerebellum of control rats as well as those treated with *N. oculata* extract and gold nano-extract (Fig. 6). Rats injected with Li_2_CO_3_ displayed nuclear pyknosis and degeneration in certain purkenji cells (arrow-pn). The neurons in the cerebellum of Li_2_CO_3_-intoxicated rats treated with *N. oculata* extract and gold nano-extract have normal histological structures (arrow-n). The histopathological scores in Supplementary Table 5 show that the cerebellum of rats injected with Li_2_CO_3_ had the most severe damage (25–50%). However, this damage was significantly reduced to 0–25% after treatment with *N. oculata* extract and gold nano-extract.

### Electrophoretic assays

#### Protein pattern

**Fig. 7:**
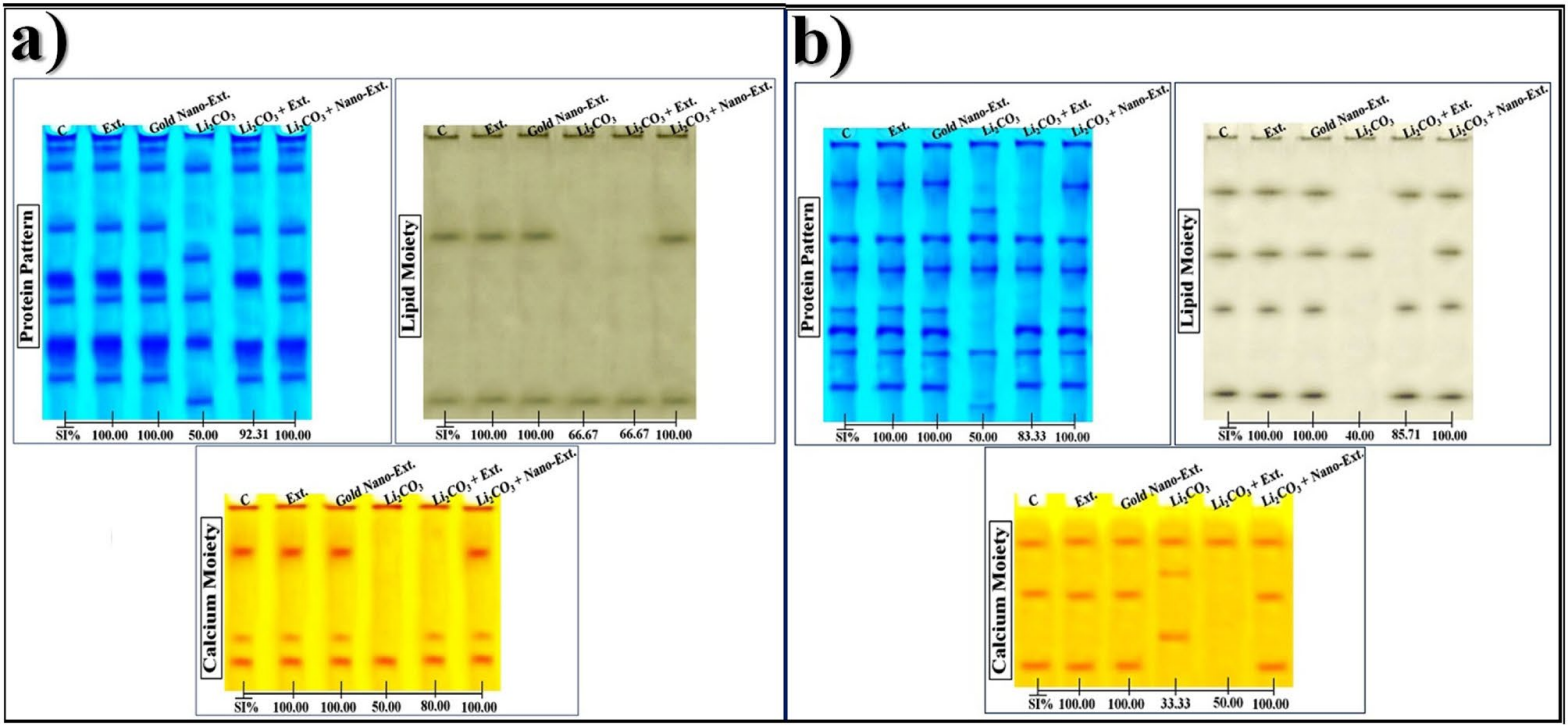
Effect of gold nanoparticles (Au-NPs) biosynthesized by *N. oculata* algal extract against the physiological alterations induced in the native protein patterns by lithium carbonate (Li_2_CO_3_) in (**a**) kidney, and (**b**) brain tissues of rats.

Data illustrated in Fig. 7a showed that 7 native protein bands (Rfs 0.04, 0.11, 0.32, 0.49, 0.57, 0.72, and 0.84; Int. 170.18, 152.07, 153.49, 183.50, 150.47, 186.66, and 159.33; B% 11.70, 13.86, 13.98, 15.77, 12.42, 20.22, and 12.05, respectively) were identified in the kidney of control rats. Two common bands (Rfs 0.11 and 0.72) were identified. When comparing the kidneys of the groups treated with *N. oculata* algal extract and gold nano-extract to the control, no differences were observed. The physiological changes were represented in the Li_2_CO_3_-injected group by the hiding of 4 normal protein bands with existing 2 abnormal (characteristic) ones (Rfs 0.42 and 0.92; Int. 158.32 and 158.20; B% 17.40 and 16.63, respectively). Therefore, the physiological similarity of this group to the control decreased to 50.00% (GD = 50.00%).

The native protein pattern was improved by the native algal extract, which obscured the unique bands and restored the four missing normal ones identified at Rfs 0.05, 0.33, 0.49, and 0.84 (Int. 173.30, 154.15, 176.04, and 161.57; B% 14.56, 15.11, 19.72, 15.19, respectively). As a result, this group’s resemblance to the control rose to 92.31% (GD = 7.69%) when compared to the group that received Li_2_CO_3_ injection. The gold algal nano-extract significantly enhanced the native protein pattern by restoring the four missing normal bands identified at Rfs 0.05, 0.33, 0.49, and 0.83 (Int. 169.66, 155.33, 183.81, and 158.72; B% 10.96, 11.23, 21.77, and 11.72, respectively) and concealing the unique bands. This group is completely resemble to the control (SI = 100.00%; GD = 0.00%) at the physiological state.

In the brain tissue of the control rats, the native protein pattern was represented by 7 bands (Rfs 0.15, 0.34, 0.45, 0.60, 0.67, 0.75, and 0.87; Int. 165.23, 156.06, 171.40, 150.87, 177.82, 160.40, and 152.66; B% 15.83, 11.63, 13.86, 9.31, 15.90, 13.32, and 20.15, respectively) (Fig. 7b). Three common bands (Rfs 0.34, 0.45, and 0.75) were identified. No variations were noticed in brains of the native algal extract and gold nano-extract treated groups compared to control. The physiological changes were represented in the Li_2_CO_3_-injected group by the hiding of 4 normal protein bands with existing 2 abnormal (characteristic) ones (Rfs 0.24 and 0.94; Int. 150.01 and 137.91; B% 15.60 and 13.20, respectively). As a result, this group’s physiological resemblance to the control dropped to 50.00% (GD = 50.00%).

The native protein pattern was improved slightly by the native algal extract, which obscured the unique bands and restored only one missing normal one identified at Rf 0.67 (Int. 183.88; B% 19.50). As a result, this group’s resemblance to the control rose to 83.33% (GD = 16.67%) when compared to the group that received Li_2_CO_3_ injection. The gold algal nano-extract significantly enhanced the native protein pattern by restoring the three missing normal bands identified at Rfs 0.16, 0.60, and 0.87 (Int. 166.98, 149.81, and 158.55; B% 14.80, 11.46, and 17.24, respectively) and concealing the unique bands. This group is completely resemble to the control (SI-100.00%; GD = 0.00%) at the physiological state.

#### Lipid moiety of native protein pattern

In the kidney tissue of control rats (Fig. 7a), the lipid moiety of the protein pattern was represented by 2 bands (Rfs 0.34 and 0.90; Int. 121.74 and 109.04; B% 52.75 and 47.25, respectively). There was only one common band found (Rf 0.90). No characteristic bands were observed. The physiological changes in the Li_2_CO_3_-injected group were represented by obscuring one normal band. The physiological similarity of this group to the control group decreased to 66.67% (GD = 33.33%).

The native algal extract could not improve this protein pattern. As a result, this group is as comparable to the control as the Li_2_CO_3_-injected group (SI = 66.67%; GD = 33.33%). The gold nano-extract improved this native protein pattern by restoring back the missing (normal) band at Rf 0.35 (Int. 128.17; B% 51.90). As a result, at the qualitative level, this treated group became identical to the control (SI = 100.00%; GD = 0.00%).

In the brain tissue of the control rats (Fig. 7b), the native pattern was represented by 4 bands (Rfs 0.19, 0.41, 0.59, and 0.89; Int. 69.39, 70.47, 74.32, and 88.12; B% 26.11, 25.04, 21.23, and 27.63, respectively). No common or characteristic bands were observed. The Li_2_CO_3_ injection caused severe changes represented by hiding three normal bands. Therefore, the physiological similarity of this group to the control group decreased to 40.00% (GD = 60.00%).

The native algal extract improved this pattern slightly by restoring the three missing bands (Rfs 0.20, 0.59, and 0.90; Int. 78.73, 72.40, and 88.01; B% 31.62, 31.26, and 37.12, respectively). As a result, this group’s resemblance to the control rose to 85.71% (GD = 14.29%) when compared to the Li_2_CO_3_-intoxicated group. The gold nano-extract improved this native protein pattern by restoring back the missing (normal) bands at Rfs 0.20, 0.58, and 0.90 (Int. 69.89, 83.74, and 77.49; B% 26.18, 19.07, and 29.60, respectively). As a result, at the physiological level, this treated group became identical to the control by 100.00% (GD = 0.00%).

#### Calcium moiety of native protein pattern

In the kidney tissue of the control group (Fig. 7a), the calcium moiety of the native pattern was represented by 3 bands (Rfs 0.24, 0.72, and 0.85; Int. 91.18, 67.66, and 80.30; B% 44.94, 23.02, and 32.04, respectively). Only one common band (Rf 0.85) was identified. There were no changes between the kidneys of the groups treated with native algal extract and gold nano-extract and the control group. The physiological abnormalities were represented in the Li_2_CO_3_-injected group by obscuring 2 normal bands. Therefore, the physiological similarity of this group to the control group decreased to 50.00% (GD = 50.00%).

A slight improvement was noticed in the native algal extract treated group by restoring only one absent (normal) band (Rf 0.71; Int. 68.80; B% 43.18). Therefore, this group was qualitatively similar to the control group by 80.00% (GD = 20.00%). This native pattern was ameliorated highly by the gold nano-extract by restoring the missing (normal) bands completely (Rfs 0.25 and 0.70; Int. 91.35 and 68.75; B% 41.07 and 25.36, respectively) in the treated group, which became completely resemble to the control (SI = 100.00%; GD = 0.00%).

In the brain tissue of the control rats (Fig. 7b), the native pattern was represented by 4 bands (Rfs 0.19, 0.48, and 0.88; Int. 82.31, 82.77, and 85.41; B% 31.34, 32.42, and 36.24, respectively). Only one common band (Rf 0.19) was identified. In the Li_2_CO_3_-injected group, the physiological abnormalities were represented by 2 normal bands with existing 2 abnormal (characteristic) ones identified at Rfs 0.36 and 0.72 (Int. 72.52 and 78.96; B% 24.17 and 38.47, respectively). The physiological similarity of this group to the control group decreased severely to 33.33% (GD = 66.67%).

This native pattern was ameliorated slightly by the native algal extract through concealing the abnormal bands. Therefore, this group became physiologically similar to the control group by 50.00% (GD = 50.00%). This native pattern was ameliorated highly by the gold nano-extract by restoring the missing (normal) bands completely (Rfs 0.49 and 0.89; Int. 82.38 and 84.56; B% 32.29 and 35.83, respectively) in the treated group, which became qualitatively similar to the control by 100.00% (GD = 0.00%).

#### Catalase (CAT) pattern

**Fig. 8:**
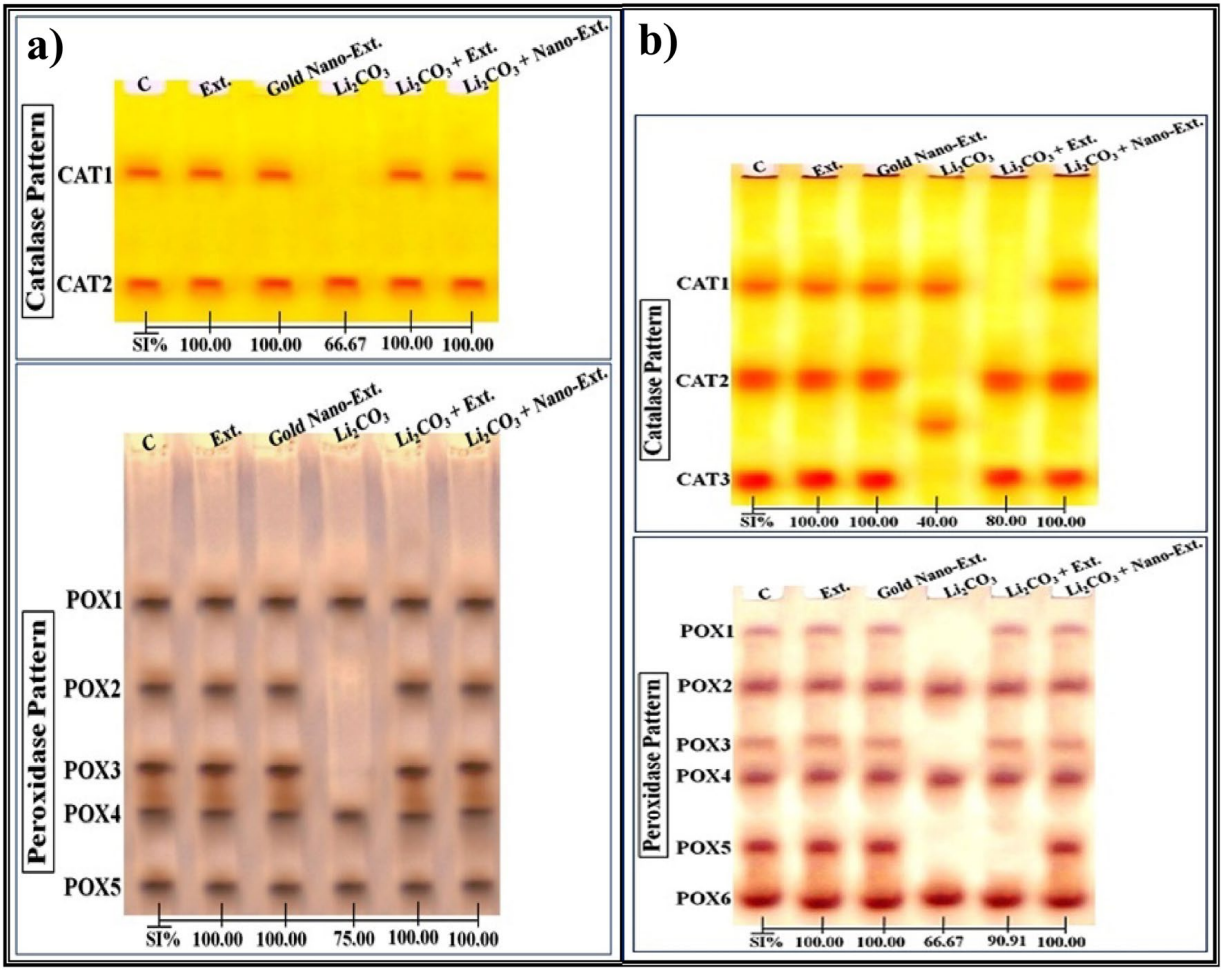
Effect of gold nanoparticles (Au-NPs) biosynthesized by *N. oculata* algal extract against the physiological alterations induced in the electrophoretic antioxidant isoenzymes patterns by lithium carbonate (Li_2_CO_3_) in (**a**) kidney, and (**b**) brain tissues of rats.

The CAT isoenzyme pattern was represented in the kidney tissue of the control rats by 2 types (Rfs 0.35 and 0.85; Int. 73.64 and 99.74; B% 59.89 and 40.11, respectively) (Fig. 8a). The 2nd CAT type (CAT2) is considered common band. No characteristic bands were observed. There were no changes between the kidneys of the groups treated with native algal extract and gold nano-extract and the control group. In the Li_2_CO_3_ intoxicated group, the qualitative abnormalities were represented by obscuring the 1st CAT type (CAT1). The physiological similarity in this group decreased to 66.67% (GD = 33.33%) compared to the control group.

In the native algal extract and gold nano-extract treated groups, the CAT isoenzyme pattern was improved by reintroducing the missing normal (CAT1) type identified at Rf 0.35 (Int. 97.04; B% 41.38), which was 100.00% (GD = 0.00%) closer to the physiological control.

In the brain tissue of the control group, the isoenzyme pattern was represented by three CAT types (Rfs 0.32, 0.58, and 0.88; Int. 95.08, 114.20, and 116.23; B% 32.04, 24.70, and 43.26, respectively) (Fig. 8b). No common bands were identified. In the Li_2_CO_3_ injected group, the physiological changes were represented by hiding two normal CAT types (CAT2 and CAT3) with the appearance of one abnormal (characteristic) band (Rf 0.72; Int. 89.40; B% 47.77). The physiological similarity of this group to the control group decreased to 40.00% (GD = 60.00%).

This isoenzyme pattern was ameliorated slightly by the native algal extract through concealing the abnormal band with restoring only one missing CAT type (CAT2) identified at Rf 0.59 (Int.112.11; B% 50.45). Therefore, this group became physiologically similar to the control group by 80.00% (GD = 20.00%). This isoenzyme pattern was ameliorated highly by the gold nano-extract through concealing the abnormal band with restoring the missing CAT types (CAT2 and CAT3) identified at Rfs 0.59 and 0.88 (Int. 122.94 and 101.76; B% 22.16 and 42.98, respectively). As a result, this group became qualitatively similar to the control by 100.00% (GD = 0.00%).

#### Peroxidase (POX) pattern

The POX isoenzyme pattern in the kidney tissue of the control group was represented by 5 types (Rfs 0.32, 0.50, 0.66, 0.76, and 0.91; Int. 144.11, 150.27, 163.07, 133.23, and 130.03; B% 18.23, 16.63, 15.47, 23.96, and 25.70, respectively) **(**Fig. 8a**)**. Three POX types (POX1, POX4, and POX5) are considered common bands. No characteristic bands were observed. The qualitative alterations were represented by the disappearance of two normal POX types (POX2 and POX3) in the Li_2_CO_3_-injected group, which became physiological similar to the control group by 75.00% (GD = 25.00%).

The deleterious effect induced by Li_2_CO_3_ injection was highly alleviated by restoring the two absent (normal) POX types (POX2 and POX3 types) identified at Rfs 0.50 and 0.67 in the native algal extract (Int. 138.85 and 167.41; B% 25.18 and 9.43, respectively) and gold nano-extract treated groups (Int. 146.30 and 150.30; B% 22.47 and 12.11, respectively). Both treated groups became completely resemble the control (SI = 100.00%; GD = 0.00%) at the physiological state.

In the brain tissue of the control rats, the isoenzyme pattern was represented by 6 types (Rfs 0.10, 0.26, 0.43, 0.53, 0.73, and 0.89; Int. 65.93, 84.54, 76.33, 101.98, 128.45, and 119.43; B% 7.16, 20.40, 9.21, 19.07, 18.21, and 25.94, respectively) (Fig. 8b). Four POX types (POX2, POX4, and POX6) are considered common bands. No characteristic bands were identified. The physiological changes in the Li_2_CO_3_ intoxicated group were represented in this isoenzyme pattern by concealing 3 normal POX types (POX1, POX3, and POX5 types). As a result, this group had the lowest SI value (SI = 66.67%; GD = 33.33%) when compared to the control group.

The adverse effect induced by Li_2_CO_3_ intoxication was diminished slightly by the native algal extract through restoring two absent normal POX types (POX1 and POX3) (Rfs 0.09 and 0.43; Int. 65.10 and 75.04; B% 9.66 and 12.25, respectively). This restoration brought the levels back to 90.91% similarity (GD = 9.09%) with the control group at the physiological level. The three missing normal POX types (POX1, POX3, and POX5) (Rf 0.09, 0.43, and 0.73; Int. 60.77, 76.77, and 120.74; B% 8.53, 9.37, and 19.53, respectively) were restored in the gold nano-extract treated group, resulting in a significant improvement. As a result, this group and the control group were identical (SI = 100.00%; GD = 0.00%).

#### Esterase (EST) pattern

**Fig. 9:**
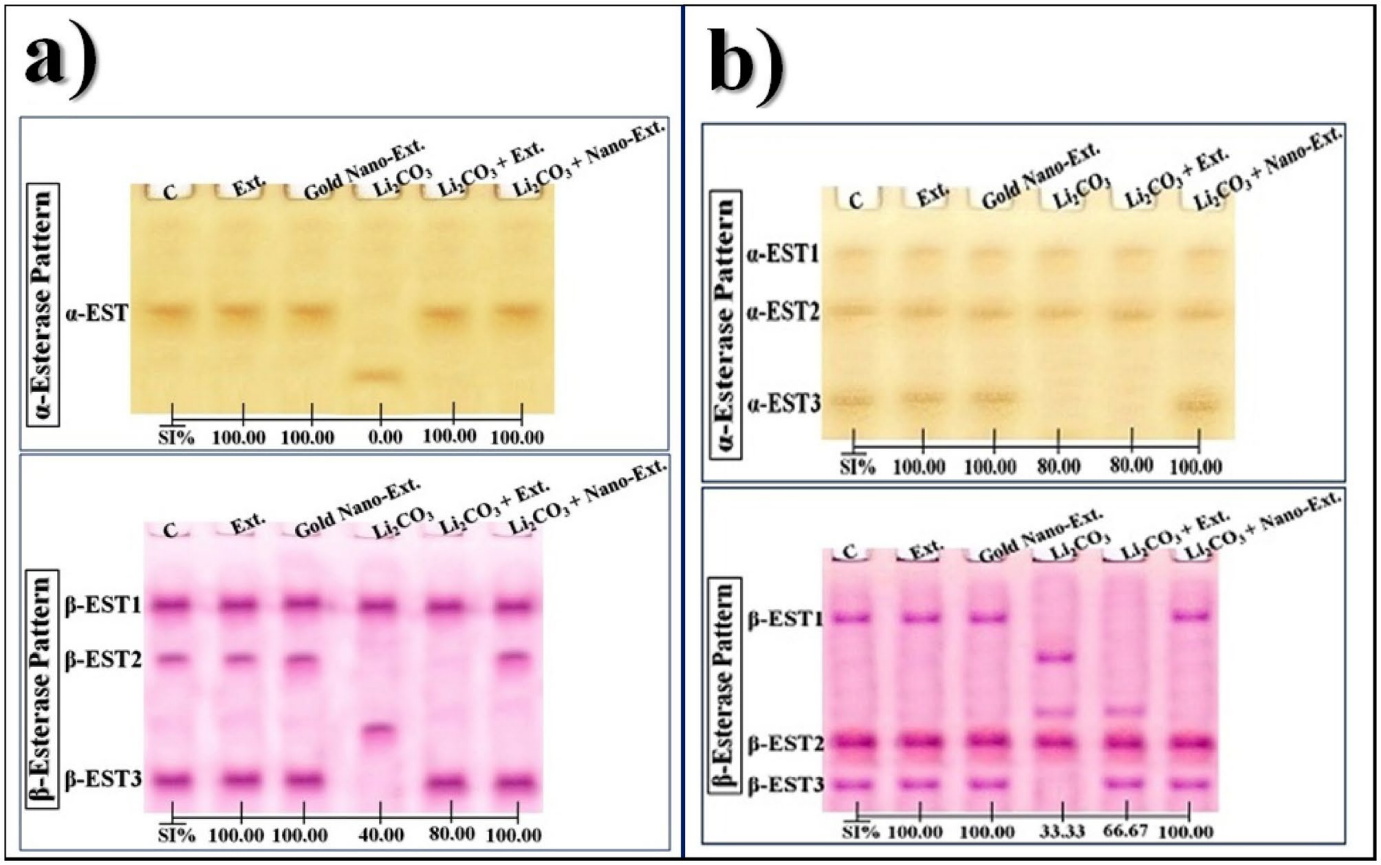
Effect of gold nanoparticles (Au-NPs) biosynthesized by *N. oculata* algal extract against the physiological alterations induced in the electrophoretic α- and β- esterase (EST) isoenzymes patterns by lithium carbonate (Li_2_CO_3_) in (**a**) kidney, and (**b**) brain tissues of rats.

The electrophoretic α-EST isoenzyme pattern in the kidney tissue of the control group was represented by only one type (Rf 0.53; Int. 72.63; B% 100.00) (Fig. 9a). No common bands were identified. No differences were observed in native algal extract and gold nano-extract treated groups compared to the kidneys of the control rats. The Li_2_CO_3_ injection caused qualitative alterations in the isoenzyme pattern, resulting in the disappearance of the normal α-EST type and the appearance of a characteristic (abnormal) band (Rf 0.83; Int. 60.91; B% 100.00). As a result, there is no physiological similarity to the control group (SI = 0.00%; GD = 100.00%).

The native algal extract and gold nano-extract restored the physiological state of the α-EST isoenzyme pattern by hiding the characteristic band and reintroducing the missing normal (α-EST) type identified at Rf 0.54 (Int. 72.84; B% 100.00). Therefore, these groups completely resemble the control (SI = 100.00%; GD = 0.00%).

The isoenzyme pattern in the brain tissue of the control group was represented by three types (Rfs 0.19, 0.44, and 0.84; Int. 47.98, 57.99, and 70.29; B% 28.48, 41.04, and 30.48, respectively) (Fig. 9b). The 1st and 2nd α-EST types (α-EST1 and α-EST2) are considered common bands. No characteristic bands were detected. The qualitative changes in the isoenzyme pattern in the Li_2_CO_3_-injected group were represented by the obscuring of the 3rd α-EST type (α-EST3). Therefore, the physiological similarity of this group to the control group decreased to 80.00% (GD = 20.00%).

The native algal extract could not improve this isoenzyme pattern. As a result, this group is as comparable to the control as the Li_2_CO_3_-injected group (SI = 80.00%; GD = 20.00%). The gold nano-extract improved this isoenzyme pattern by restoring back the missing α-EST type (α-EST3) at Rf 0.86 (Int. 72.45; B% 24.36). As a result, at the physiological level, this treated group resembled the control group completely (SI = 100.00%; GD = 0.00%).

In the kidney tissue of the control group, the β-EST isoenzyme pattern was represented by three types (Rfs 0.24, 0.42, and 0.85; Int. 81.63, 75.38, and 90.23; B% 41.96, 23.93, and 34.10, respectively) (Fig. 9a). The 1st β-EST type (β-EST1) is considered a common band. No changes were detected in native algal extract and gold nano-extract treated groups compared to the control group. The physiological alterations were represented by the disappearance of the 2nd and 3rd normal β-EST types (β-EST2 and β-EST3) with the appearance of one characteristic band (Rf 0.67; Int. 76.72; B% 46.62) in the Li_2_CO_3_-injected group, which exhibited the lowest physiological similarity to the control group (SI = 40.00%; GD = 60.00%).

The isoenzyme pattern was slightly improved by hiding the characteristic band and restoring only one of the missing types (β-EST3) (Rf 0.86; Int. 93.38; B% 46.88) in the algal extract treated group. As a result, this group’s resemblance to the control increased to 80.00% (GD = 20.00%) when compared to the group that received Li_2_CO_3_ injection. The two missing normal β-EST types (β-EST2 and β-EST3) (Rfs 0.42 and 0.86; Int. 72.80 and 92.94; B% 31.52 and 35.04, respectively) were restored in the gold nano-extract treated group, resulting in a significant improvement. Consequantly, this group and the control group were identical (SI = 100.00%; GD = 0.00%).

In the brain tissue of the control group, the isoenzyme pattern was represented by three types (Rfs 0.23, 0.71, and 0.87; Int. 91.10, 101.50, and 83.24; B% 21.02, 53.95, and 25.03, respectively) (Fig. 9b). The β-EST2 type is considered a common band. Only one characteristic band was identified in the Li_2_CO_3_-injected group at Rf 0.38 (Int. 84.92; B% 28.25). The injection of Li_2_CO_3_ caused physiological abnormalities, as evidenced by the disappearance of two normal types (β-EST1 and β-EST3) and the appearance of two abnormal bands (Rfs 0.38 and 0.59; Int. 84.92 and 79.27; B% 28.25 and 18.58, respectively). Consequently, the lowest SI value (SI = 33.33%; GD = 66.67%) was observed compared to the control group.

The adverse effect induced by Li_2_CO_3_ intoxication was diminished slightly by the native algal extract through the disappearance of the characteristic band and restoring only one normal EST type (EST3) (Rf 0.87; Int. 84.08; B% 29.26). This restoration brought the levels back to 66.67% similarity (GD = 33.33%) with the control group at the physiological level. The gold nano-extract highly improved the isoenzyme pattern by eliminating the two abnormal bands and restoring the missing β-EST1 and β-EST3 types (Rfs 0.11 and 0.87; Int. 86.21 and 82.51; B% 24.08 and 26.51, respectively). As a result, the isoenzyme pattern closely resembled that of the control group (SI = 100.00%; GD = 0.00%).

### Molecular assay

**Fig. 10:**
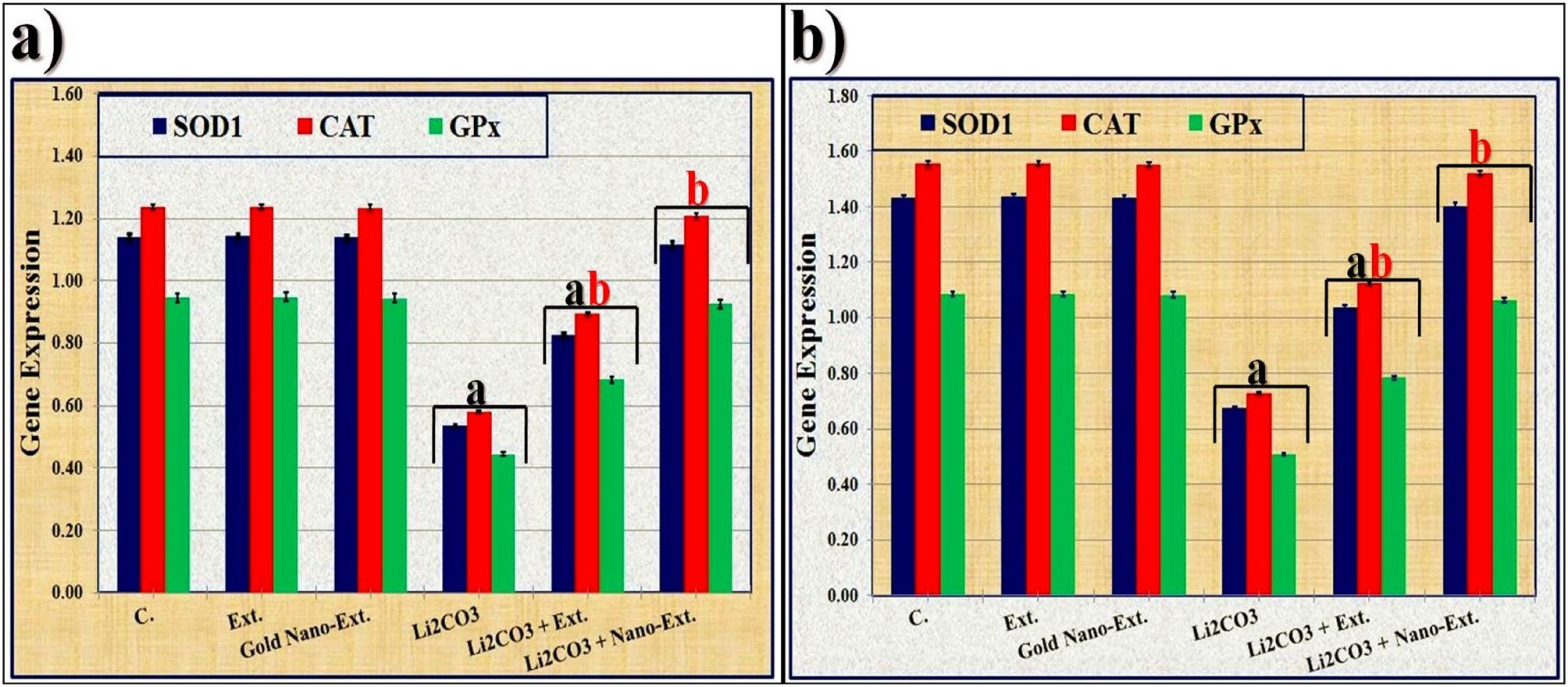
Effect of gold nanoparticles (Au-NPs) biosynthesized by *N. oculata* algal extract against the changes induced by lithium carbonate (Li_2_CO_3_) in levels of mRNA expression of antioxidants enzymes (SOD1, CAT, and GPx1) in (**a**) kidney and (**b**) brain in rats. Data were calculated from five replicates and expressed as mean ± SE, (**a**) significant versus control group, (**b**) significant versus toxic (Li_2_CO_3_) group at *p* ≤ 0.05.

The study focused on quantifying the mRNA expression levels of antioxidant enzymes (SOD1, CAT, and GPx1) and inflammation markers (NF-κb, IL-6, and IL-1β) in kidney and brain tissues. Figure 10 shows that Li_2_CO_3_ significantly (*p* ≤ 0.05) decreased the mRNA expression levels of antioxidant enzymes (SOD1, CAT, and GPx) in rat kidney and brain tissues compared to the control group. Treatment with *N. Oculata* extract significantly (*p* ≤ 0.05) increased the mRNA expression levels of antioxidant enzymes compared to the Li_2_CO_3_-intoxicated group but did not totally restore them to normal values. In the gold nano-extract treated group, mRNA expression levels of antioxidant enzymes increased significantly (*p* ≤ 0.05) and were restored to normalcy.

**Fig. 11:**
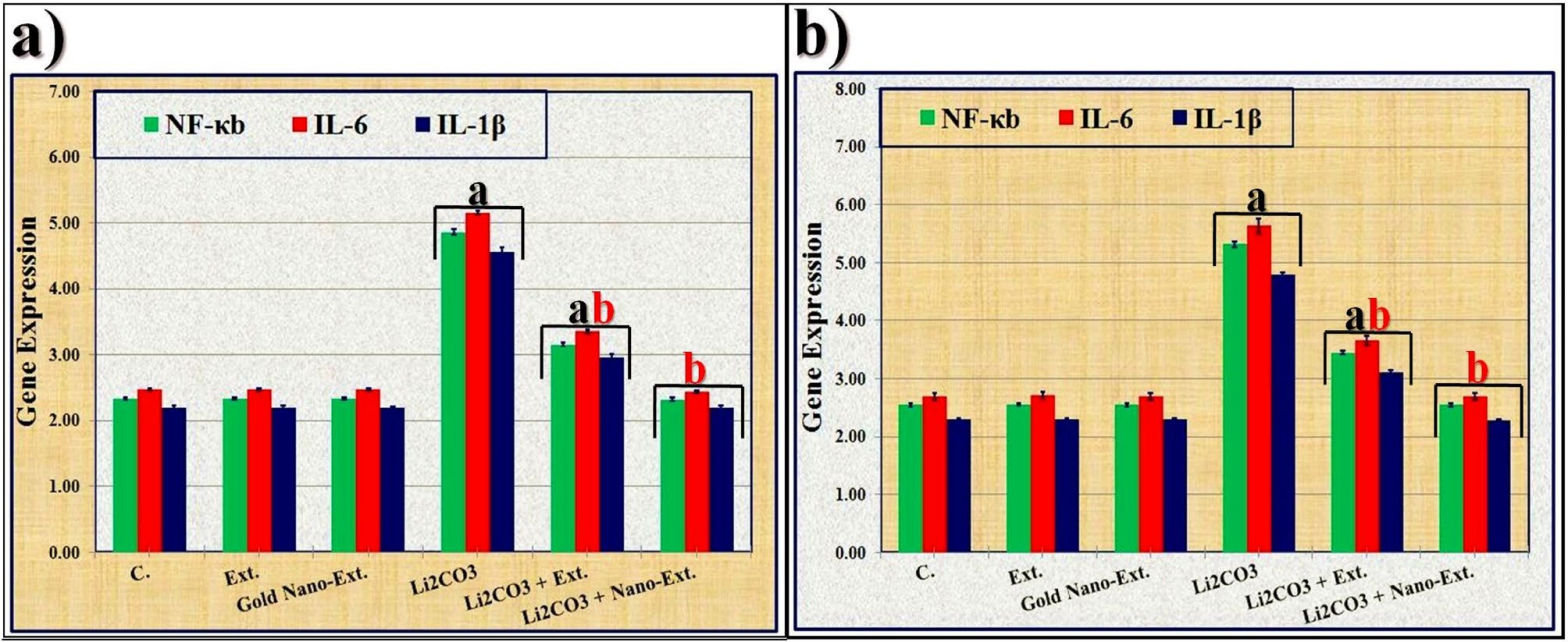
Effect of gold nanoparticles (Au-NPs) biosynthesized by *N. oculata* algal extract against the changes induced by lithium carbonate (Li_2_CO_3_) in levels of mRNA expression of inflammation markers (NF-κb, IL-6, and IL-1β) in (**a**) kidney and (**b**) brain in rats. Data were calculated from five replicates and expressed as mean ± SE, (**a**) significant versus control group, (**b**) significant versus toxic (Li_2_CO_3_) group at *p* ≤ 0.05.

In the Li_2_CO_3_-injected group, mRNA levels of NF-κb, IL-6, and IL-1β were significantly (*p* ≤ 0.05) higher in kidney and brain tissues compared to the control group (Fig. 11). Treatment with *N. oculata* extract significantly (*p* ≤ 0.05) reduced the mRNA expression levels of these indicators compared to the Li_2_CO_3_-intoxicated group, but did not fully restore them to normal levels. In contrast, treatment with the gold nano-extract restored these values to normal levels.

## Discussion

Lithium medications, such as Li_2_CO_3_, are effective in treating BDs but can cause Li intoxication due to its narrow therapeutic range^56^. The accumulation of Li salts in renal tubular cells can lead to nephrotoxic effects. Li also affects the anti-diuretic function of vasopressin, causing polydipsia and polyuria^57^. In rats, long-term Li administration alters receptor sensitivity and neurotransmitter systems, including serotonin and dopamine. It may also influence dopamine levels by modifying receptor expression and release in response to stimuli^58^.

The study aims to evaluate the efficiency of Au-NPs synthesized using *N. oculata* extract against Li_2_CO_3_-induced pathologies in animal models. Hematological measurements can help assess the impact of chemical compounds or plant extracts on blood parameters and detect any adverse effects^59^. The study found a significant increase in white blood cells (WBCs) and their differential count in the Li_2_CO_3_-injected group compared to the control group. This increase may be attributed to lithium’s stimulation of the adrenal cortex, leading to leukocytosis^60^. Additionally, Li may influence bone marrow by promoting the differentiation of stem cells into granulocytes^61^.

Recent studies have indicated that Li_2_CO_3_ can lead to liver damage, as observed in the current study. Li was found to elevate levels of liver enzymes (ALP, AST, ALP, and GGT), which are commonly used as indicators of liver function. This is consistent with the findings of Mehana et al.^62^, who linked liver damage from Li_2_CO_3_ to reactive electrophiles that cause necrosis and membrane damage in liver tissue. Consequently, cytoplasmic enzymes may leak into the bloodstream due to increased membrane permeability during the initial stages of liver damage.

 Li can have physiological effects on the kidneys, leading to nephrotoxicity, which damages the renal tubules and reduces concentration^63^. In its carbonate form, Li increases the selective permeability of sodium and Li by forming ion pairs with sodium^64^. Moreover, Li can displace sodium or potassium in cells, causing imbalances in salt, water, and electrolytes^65^.

According to Mesallam et al.^66^, who reported that the concentration of nitrogenous compounds increased due to renal injury and nephrotoxicity induced by Li_2_CO_3_, the current study found that levels of renal markers (urea, creatinine, and BUN) increased significantly. Weiner et al.^67^ found that the accumulation of Li in the distal regions of the nephron through the epithelial sodium channel led to a decrease in blood uric acid, total protein, and albumin levels. The decrease in total protein and albumin levels suggests that the kidneys were unable to reabsorb these components. Since the liver is responsible for producing plasma proteins, they must pass through glomerular filtration^68^ and may be affected by liver injury, leading to reduced protein synthesis, digestion, and absorption^69^. The study found that Li_2_CO_3_ intoxication led to a significant decrease in HDL-c levels and an increase in cholesterol, TGs, and LDL-c levels compared to the control group. This could be due to lithium’s interaction with the -SH group in the active site of the lipoprotein lipase enzyme, which plays a crucial role in lipid processing, transport, and tissue uptake^70^. The levels of heart enzymes (CK and LDH) in the blood of the Li_2_CO_3_-intoxicated group increased. This was caused by Li affecting sodium transporter channels in neurons, which in turn altered catecholamine metabolism and storage. Additionally, Li disrupted sodium/calcium exchangers and sodium/potassium pumps, leading to abnormalities in the cardiac cell membrane physiology. This resulted in hypercalcemia and hypokalemia, which affected the propagation of electrical impulses and depolarization in the heart^71^.

Aboulthana et al.^14^ found that *N. oculata* algal extract administration normalized impaired biochemical measurements. This improvement is attributed to the extract’s phenolics, chlorophylls, and carotenoids, which possess antioxidant properties and protect liver, kidney, and heart tissues from damage by reactive oxygen and nitrogen species. The algal extract’s polysaccharides reduce cholesterol and triglyceride levels by limiting intestinal absorption^72^. Furthermore, eicosapentaenoic and docosahexaenoic fatty acids in the extract lower LDL-c and raise HDL-c levels^73^. Synthesizing Au-NPs from the algal extract enhanced its biological efficiency, as demonstrated by Bekheit et al.^30^. The gold algal nano-extract preserved cell membrane integrity against reactive species and restored impaired hepatic, renal, and cardiac markers to normal values.

The Li_2_CO_3_ intoxication led to notable alterations in antioxidant markers in both kidney and brain tissues, resulting in decreased levels of enzymatic (SOD, CAT, and GPx) and non-enzymatic antioxidants (TAC and GSH). This aligns with findings from a study by Joshi et al.^74^, indicating that these changes may be attributed to heightened oxidative stress, causing an excessive consumption of antioxidants to counteract the effects of peroxidation reactions. Toplan et al.^75^ suggested that decreased antioxidants in tissues indicate high levels of oxidative stress, leading to increased production of endogenous H_2_O_2_, resulting in elevated levels of the peroxidation products (LPO and TPC)  as observed in the current study. Glutathione (GSH) is an important marker for assessing tissue damage caused by oxidative toxins, as it plays a crucial role in the intracellular protective system. Lipid peroxidation leads to the consumption of glutathione by glutathione-related enzymes for detoxifying peroxides^76^. Therefore, an increase in peroxidation products is typically accompanied by a depletion of GSH.

The treatment with native algal extract significantly increased markers of the antioxidant system compared to the Li_2_CO_3_-intoxicated group. This aligns with El-Feky et al.^77^, who suggested that the algal extract is rich in phenolics, chlorophylls, carotenoids, and bioactive peptides. The presence of flavonoids, pyrogallol, catechin, and four phenolic compounds (cinnamic acid, *p*-coumaric acid, *p*-hydroxybenzoic acid, and gallic acid) contributes to strong antioxidant and scavenging properties, reducing oxidized intermediates during peroxidation processes . By significantly increasing the antioxidants and reducing the peroxidation products, the administration of the gold nano-extract improved the antioxidant status and returned their levels to normal. This result is consistent with Aboulthana et al.^78^, who demonstrated that Au-NPs activate the protective system against oxidative disruptions by enhancing the activity of antioxidant enzymes. The gold nano-extract reported higher antioxidant activity than the plant extract alone because the antioxidant compounds from plant extracts are adsorbed onto the active surface of the Au-NPs^79^. The surface reaction and the high surface area-to-volume ratio of the Au-NPs may also influence the interaction and scavenging activity of free radicals, resulting in a higher antioxidant activity^80^.

The inflammatory effect of Li_2_CO_3_ had a negative impact on the cellular defense system, leading to cytokine-mediated inflammation. This resulted in a significant increase in levels of the proinflammatory cytokines TNF-α and IL-6 in both kidney and brain tissues^81^. The elevation in the levels of these measurements may be linked to influence of Li_2_CO_3_ on regulating miR-29 expression^82^. Pacholko and Bekar^83^ also noted that the Li_2_CO_3_-injected group had higher levels of TNF-α because Li stimulates TNF-α, which may help induce granulocytosis. Thus, by increasing TNF-α-induced IL-6 production in fibrosarcoma cells, Li_2_CO_3_ can also increase IL-6 secretion^84^. The treatment with native algal extract reduced levels of these inflammatory markers compared to the Li_2_CO_3_-injected group. This might be attributed to the presence of eicosapentaenoic acid and omega-3 polyunsaturated fatty acids, which are well known for their inhibitory effect on inflammatory reactions, leading to a decrease in the production of these pro-inflammatory cytokines. Therefore, it improves the integrity and function of the tissues^85^. Due to the presence of Au-NPs, which have immunomodulatory effects by inhibiting the expression of nuclear factor kappa B (NF-κB) transcription factors and reducing subsequent cellular responses and pro-inflammatory cytokine production, the nano-extract treatment restored normal levels of these proinflammatory markers^86^. Additionally, Rizwan et al.^87^ showed that the presence of Au-NPs increased the anti-inflammatory activity by altering antioxidant defense systems.

Acetylcholine (ACh) is broken down by the enzyme AChE in cholinergic synapses and neuromuscular junctions. AChE is essential for promoting apoptosis, and reducing its expression can prevent apoptosis^88^. In this study, the activity of AChE decreased in the kidney and brain tissues of the Li_2_CO_3_-injected group, consistent with Rakha et al.^89^ showing that Li reduces AChE activity by blocking dopaminergic transmission. This imbalance between dopamine and ACh can lead to hyperactivity. The gold nano-extract had a stronger inhibitory effect on AChE activity compared to the native algal extract, potentially due to its high binding affinity to ChEs. This suggests that gold nanoparticles can restore AChE activity to normal levels^90^.

Histopathological examination at the end of the study revealed deformative changes caused by Li , including damage to the epithelial lining of the glomerulus in renal tissue without cellular growth. This finding supports previous research by Ommati et al.^91^, indicating that oxidative stress induced by ROS may contribute to renal tissue damage. ROS can interact with cell macromolecules, leading to cellular-level organ failure. Rats treated with Li also showed significant histopathological alterations such as tubular degeneration, glomerular dilatation, interstitial inflammation, and hemorrhage, as reported by Badawy et al.^92^.

It was noticed that Li also caused structural and morphological changes in the brain tissue, as evidenced by nuclear pyknosis and degeneration in the cerebral cortex, hippocampus neurons, and cerebellar Purkinje cells. This is consistent with the hypothesis of Nasir and Jaffat^93^ that the cerebellum contains more Li than other organs, followed by the cerebrum and the kidneys. Furthermore, Li stimulates the growth of bone marrow and neural stem cells in the striatum, forebrain, and subventricular zone, increasing the density and volume of brain cells^94^. The histopathological lesions identified in the Li-intoxicated group were lessened after receiving therapy with native algal extract. This may be explained by the active ingredients’ metal-chelating properties, which allow the algal extract to reduce Li accumulation and consequently the histopathological lesions caused by oxidative stress and excessive ROS generation^95^. Additionally, eicosapentaenoic acid and omega-3 polyunsaturated fatty acids bind to immune cells and play a crucial role in immune system activation^96^. The gold nano-extract restored the tissues to their normal architecture, similar to the control group. This aligns with findings of Saleh et al.^97^, suggesting that the therapeutic potential of Au-NPs may be attributed to their antioxidant properties, which increase antioxidant levels, decrease reactive oxygen levels, and reduce oxidative damage.

Electrophoretic techniques can be used to identify variations in physical and chemical characteristics in animals exposed to different conditions^98^. The percentage of similarity index (SI) can serve as a valuable tool in analyzing electrophoretic protein and isoenzyme patterns to assess the physiological status of the animal. Elevated SI values indicate a close resemblance in band numbers and arrangements between the two samples^99^.

Li caused physiological alterations in the kidney and brain tissues, which were detected by electrophoresis of the native proteins, lipids, and calcium moieties of the native proteins. This is in line with research by Kehm et al.^100^, which showed that certain native proteins are susceptible to reactive species that can oxidize structural proteins and inhibit the proteolytic system, changing the structure of proteins. This results in a number of downstream consequences, such as decreased fidelity of damaged DNA polymerases during DNA replication, altered cell absorption, inactivation of DNA repair enzymes, and inhibition of enzymatic and binding activities. These electrophoretic alterations could impact the amounts, locations, or translation of RNA transcripts^101^. Furthermore, alterations in native protein composition and pattern may be linked to modifications in DNA organization, which may result in modifications to DNA activity and protein synthesis. Antioxidant enzymes are thought to be tissue-dependent components of the antioxidant system. Their behavior varies according to the tissue. The changes in the antioxidant system could be due to the ROS attack that targets protein contents and alters metabolic pathways^102^. A broad family of enzymes called esterases is employed as a prognostic indicator for a number of chronic diseases^103^. According to Hussien and Omar^104^, these lysosomal lipolytic enzymes promote the hydrolysis of neutral lipid ester linkages, which are found in lipid deposits and lipoprotein components, and their subsequent cleavage into the appropriate carboxylic acids.

Li led to anomalies in the electrophoretic antioxidant isoenzymes in the present investigation. This agrees with Aboulthana et al.^105^, who proposed that variations in the fractional activity of certain isoenzymes appeared to be connected with variations in the rate of protein production as a result of ROS-induced DNA damage. The protein part of native enzymes undergoes structural modifications, resulting in changes in enzymatic activity. If there were no changes in the expression of the protein, then there were no changes in enzymatic activity^106^. Additionally, binding of Li to native macromolecules caused secondary structural modifications in the enzyme, altering the electrophoretic CAT and POX patterns^107^. Furthermore, differences in glycosylated EST forms could be responsible for the unique changes in the EST pattern^108^. The normal molecular EST pattern shows a standard glycosylation pattern. However, abnormal glycosylation can affect EST stability and increase the risk of protein degradation^109^. The native algal extract contains polyphenolic compounds that enhance antioxidant and reduction activities by disrupting multiple free radical chain reactions that target biomacromolecules. This resulted in improvements in various native proteins and isoenzyme patterns^110^. When the gold nano-extract was administered, the impaired electrophoretic protein and isoenzyme patterns returned to their typical physiological state. This aligns with the hypothesis proposed by Sekar et al.^111, who demonstrated^ that the antioxidant and anti-inflammatory properties of the biosynthesized Au-NPs are responsible for protecting the macromolecules from oxidative damage.

Li significantly reduced the mRNA levels of antioxidant enzymes (SOD1, CAT, and GPx) in the rat kidneys and brains compared to the control group. It also increased the mRNA levels of inflammatory markers (NF-κb, IL-6, and IL-1β) in these tissues compared to the control group. These findings are consistent with Allagui et al.^112^ that have shown Li concentrations can down-regulate genes involved in antioxidant defenses. Additionally, Li can impact the expression of genes related to important cellular functions such as tumor suppression, transcription factors, cell signaling, oncogenes, and second messengers^113^.

The algal extract treatment, in combination with polyphenols and flavonoids, improved the mRNA levels of inflammatory and antioxidant markers, leading to an anti-inflammatory response through its antioxidant effects^114^. Supplemented *N. oculata* containing alpha-tocopherol and β-carotene enhanced antioxidant capacity by boosting the immune system^115^. The presence of Au-NPs in the gold nano-extract, known for its anti-inflammatory properties, normalized mRNA levels of inflammation markers and antioxidant enzymes, modulating the biological response at a molecular level by reducing inflammatory gene expression and increasing antioxidant gene expression^116^.

## Conclusions

Our study found that the hematological and biochemical changes caused by Li_2_CO_3_ were reversed in rats treated with gold *N. oculata* nano-extract. The levels of oxidative stress and inflammatory markers in kidney and brain tissues were normalized with the gold nano-extract treatment. Histopathological lesions from Li_2_CO_3_ intoxication were alleviated in the gold nano-extract treated group compared to the native algal extract treated group. Furthermore, the altered protein and isoenzyme patterns identified through electrophoresis returned to normal levels with the gold nano-extract treatment. Molecular analysis showed that the gold nano-extract significantly (*p* ≤ 0.05) restored the mRNA expression of antioxidant enzymes (SOD1, CAT, and GPx1) and inflammatory markers (NF-κb, IL-6, and IL-1β) affected by Li_2_CO_3_ injection, surpassing the effects of the native algal extract.

## Supplementary Information

Supplementary Information.

## Data Availability

All data obtained during this investigation are included in the publication.
