# Supplementary material for: Assessment of the biological efficacy of gold Nannochloropsis oculata nano-extract against lithium-induced toxicity in rats
Source: Sci Rep. 2026 Jan 26;16:3269. doi: 10.1038/s41598-025-33835-5 (PMC12835123; doi:10.1038/s41598-025-33835-5)
Supplement: Supplementary file 1 — Supplementary Information. [file 41598_2025_33835_MOESM1_ESM.doc]

**Supplementary Table 1** Sequences of the specific primers used for *qRT-PCR*.

| **Gene** | **Primers sequences (5' - 3')** |
| --- | --- |
| **SOD1** | **F:** CATTCCATCATTGGCCGTACT  **R:** CCACCTTTGCCCAAGTCATC |
| **CAT** | **F:** GTACAGGCCGGCTCTCACA  **R:** ACCCGTGCTTTACAGGTTAGCT |
| **GPx1** | **F:** GCGCTGGTCTCGTCCATT  **R:** TGGTGAAACCGCCTTTCTTT |
| **NF-κB** | **F:** AACTCAGTCAGGCTCCATTGC  **R:** GACAGTGCTCTCCGTCTTTCC |
| **IL-6** | **F:** AAGCCAGAGTCATTCAGAGCAA  **R:** GGTCCTTAGCCACTCCTTCT |
| **IL-1b** | **F:** AAATGCCTCGTGCTGTCTGA  **R:** CAAGGCCACAGGGATTTTGTC |
| **GAPDH** | **F:** caa ctc cca ctc ttc cac ct  **R:** gag ttg gga tag ggc ctc tc |

**Supplementary Table 2** Effect of gold nanoparticles (Au-NPs) biosynthesized by *N. oculata* algal extract against the toxicity induced by lithium carbonate (Li_2_CO_3_) on different hematological measurements in rats.

|  | | **C.** | **Algal Ext.** | **Au-Algal Nano-Ext.** | **Li_2_CO_3_** | **Li_2_CO_3_ treated with** | |
| --- | --- | --- | --- | --- | --- | --- | --- |
|  |  |  |  |  |  | **Algal Ext.** | **Au-Algal Nano-Ext.** |
| **Formed Elements** | **RBCs (**10^6^/ul**)** | 7.13 ± 0.02 | 7.24 ± 0.02 | 7.31 ± 0.02 | 7.25 ± 0.03 | 7.54 ± 0.02 | 7.08 ± 0.02 |
|  | **HB (**g/dl**)** | 13.28 ± 0.03 | 13.35 ± 0.02 | 13.19 ± 0.03 | 13.21 ± 0.04 | 13.30 ± 0.03 | 13.33 ± 0.03 |
|  | **HCT (**%**)** | 37.37 ± 0.04 | 36.95 ± 0.03 | 37.16 ± 0.04 | 36.88 ± 0.03 | 37.26 ± 0.04 | 36.89 ± 0.03 |
|  | **MCV (**um^3^**)** | 60.14 ± 0.03 | 59.76 ± 0.04 | 58.97 ± 0.03 | 59.28 ± 0.04 | 60.21 ± 0.04 | 58.87 ± 0.03 |
|  | **MCH (**pg**)** | 23.18 ± 0.02 | 22.78 ± 0.02 | 23.22 ± 0.02 | 23.24 ± 0.02 | 22.85 ± 0.02 | 22.92 ± 0.02 |
|  | **MCHC (**g/dl**)** | 35.11 ± 0.03 | 34.77 ± 0.04 | 35.24 ± 0.03 | 34.87 ± 0.04 | 35.21 ± 0.03 | 34.52 ± 0.04 |
|  | **RDW (**%**)** | 17.71 ± 0.02 | 18.04 ± 0.03 | 17.83 ± 0.03 | 18.21 ± 0.03 | 17.92 ± 0.01 | 17.59 ± 0.03 |
|  | **MPV (**um^3^**)** | 8.27 ± 0.01 | 8.58 ± 0.02 | 8.47 ± 0.01 | 8.33 ± 0.02 | 8.19 ± 0.02 | 8.37 ± 0.02 |
|  | **PLT (**10^3^/ul**)** | 433.81 ± 0.45 | 441.29 ± 0.46 | 428.75 ± 0.45 | 435.12 ± 0.41 | 442.88 ± 0.56 | 427.42 ± 0.57 |
|  | **WBCs (**10^3^/ul**)** | 7.39 ± 0.01 | 7.42 ± 0.02 | 7.37 ± 0.02 | 20.97 ± 0.02**^a^** | 11.40 ± 0.02**^ab^** | 8.43 ± 0.02**^b^** |
| **Differential Count** | **Lymp. (**10^3^/ul**)** | 5.88 ± 0.01 | 5.79 ± 0.02 | 5.77 ± 0.02 | 15.20 ± 0.02**^a^** | 9.89 ± 0.01**^ab^** | 5.72 ± 0.02**^b^** |
|  | **Mono. (**10^3^/ul**)** | 0.37 ± 0.01 | 0.34 ± 0.01 | 0.35 ± 0.01 | 1.07 ± 0.01**^a^** | 0.73 ± 0.01**^ab^** | 0.36 ± 0.01**^b^** |
|  | **Gran. (**10^3^/ul**)** | 0.22 ± 0.01 | 0.24 ± 0.01 | 0.25 ± 0.01 | 0.73 ± 0.01**^a^** | 0.51 ± 0.01**^ab^** | 0.23 ± 0.01**^b^** |

Data were calculated from five replicates and expressed as mean ± SE, **a**: significant versus control group, **b**: significant versus toxic (Li_2_CO_3_) group at *p*≤0.05.

**Supplementary Table 3** Effect of gold nanoparticles (Au-NPs) biosynthesized by *N. oculata* algal extract against the biochemical alterations induced by lithium carbonate (Li_2_CO_3_) in rats.

|  | | **C.** | **Algal Ext.** | **Au-Algal Nano-Ext.** | **Li_2_CO_3_** | **Li_2_CO_3_ treated with** | |
| --- | --- | --- | --- | --- | --- | --- | --- |
|  |  |  |  |  |  | **Algal Ext.** | **Au-Algal Nano-Ext.** |
| **Liver** | **ALT (**U/L**)** | 42.91 ± 0.01 | 43.21 ± 0.02 | 42.88 ± 0.02 | 82.09 ± 0.03**^a^** | 66.32 ± 0.02**^ab^** | 43.51 ± 0.03**^b^** |
|  | **AST (**U/L**)** | 62.22 ± 0.01 | 63.31 ± 0.02 | 62.27 ± 0.02 | 108.81 ± 0.03**^a^** | 87.67 ± 0.02**^ab^** | 64.03 ± 0.01**^b^** |
|  | **ALP (**U/L**)** | 95.79 ± 0.01 | 94.80 ± 0.02 | 95.33 ± 0.02 | 167.64 ± 0.03**^a^** | 115.71 ± 0.02**^ab^** | 93.87 ± 0.02**^b^** |
|  | **GGT (**U/L**)** | 24.15 ± 0.01 | 24.35 ± 0.02 | 25.11 ± 0.02 | 51.26 ± 0.01**^a^** | 37.69 ± 0.01**^ab^** | 24.25 ± 0.02**^b^** |
| **Kidney** | **Urea (**mg/dl**)** | 37.88 ± 0.02 | 38.15 ± 0.02 | 37.48 ± 0.02 | 94.70 ± 0.05**^a^** | 66.03 ± 0.04**^ab^** | 37.17 ± 0.02**^b^** |
|  | **Creat. (**mg/dl**)** | 1.24 ± 0.01 | 1.25 ± 0.01 | 1.27 ± 0.01 | 3.71 ± 0.02**^a^** | 2.35 ± 0.02**^ab^** | 1.26 ± 0.01**^b^** |
|  | **Uric Acid (**mg/dl**)** | 3.27 ± 0.01 | 3.22 ± 0.01 | 3.25 ± 0.01 | 1.31 ± 0.01**^a^** | 2.17 ± 0.01**^ab^** | 3.24 ± 0.01**^b^** |
|  | **BUN (**mg/dl**)** | 8.66 ± 0.03 | 8.47 ± 0.03 | 8.56 ± 0.03 | 18.26 ± 0.07**^a^** | 12.25 ± 0.05**^ab^** | 8.51 ± 0.03**^b^** |
|  | **T. Protein (**g/dl**)** | 8.16 ± 0.01 | 8.14 ± 0.01 | 8.12 ± 0.01 | 5.25 ± 0.01**^a^** | 6.86 ± 0.01**^ab^** | 8.17 ± 0.01**^b^** |
|  | **Albumin (**g/dl**)** | 3.90 ± 0.01 | 3.85 ± 0.01 | 3.84 ± 0.01 | 1.67 ± 0.01**^a^** | 2.47 ± 0.01**^ab^** | 3.91 ± 0.01**^b^** |
| **Heart** | **CK (**U/L**)** | 69.52 ± 0.02 | 70.13 ± 0.02 | 69.63 ± 0.02 | 104.28 ± 0.04**^a^** | 88.25 ± 0.04**^ab^** | 70.51 ± 0.02**^b^** |
|  | **LDH (**U/L**)** | 221.79 ± 0.08 | 224.81 ± 0.08 | 231.26 ± 0.09 | 335.69 ± 0.12**^a^** | 279.55 ± 0.13**^ab^** | 229.81 ± 0.09**^b^** |
| **Lipid Profile** | **TC (**mg/dl**)** | 88.48 ± 0.02 | 89.49 ± 0.03 | 86.57 ± 0.02 | 173.69 ± 0.04**^a^** | 127.77 ± 0.03**^ab^** | 90.29 ± 0.03**^b^** |
|  | **T.Gs (**mg/dl**)** | 101.77 ± 0.01 | 103.80 ± 0.02 | 105.64 ± 0.03 | 188.57 ± 0.04**^a^** | 147.20 ± 0.03**^ab^** | 107.07 ± 0.05**^b^** |
|  | **HDL-c(**mg/dl**)** | 18.01 ± 0.04 | 18.12 ± 0.07 | 17.92 ± 0.05 | 7.29 ± 0.08**^a^** | 10.50 ± 0.04**^ab^** | 17.89 ± 0.05**^b^** |
|  | **LDL-c(**mg/dl**)** | 50.17 ± 0.06 | 51.08 ± 0.09 | 51.23 ± 0.04 | 118.55 ± 0.04**^a^** | 87.03 ± 0.03**^ab^** | 51.24 ± 0.02**^b^** |

Data were calculated from five replicates and expressed as mean ± SE, **a**: significant versus control group, **b**: significant versus toxic (Li_2_CO_3_) group at *p*≤0.05.

**Supplementary Table 4** Histopathological score showing the effect of gold nanoparticles (Au-NPs) biosynthesized by *N. oculata* algal extract against the lesions induced by lithium carbonate (Li_2_CO_3_) in kidney tissue of rats.

| **Histopathological lesion** | **C.** | **Algal Ext.** | **Au-Algal Nano-Ext.** | **Li_2_CO_3_** | **Li_2_CO_3_ treated with** | |
| --- | --- | --- | --- | --- | --- | --- |
|  |  |  |  |  | **Algal Ext.** | **Au-Algal Nano-Ext.** |
| **Focal inflammatory cells infiltration in between the tubules** | **-** | **-** | **-** | **+++** | **++** | **-** |
| **Swelling and degeneration of the tubular lining epithelium** | **-** | **-** | **-** | **++** | **-** | **-** |

-: Nil (0-25 %), +: Mild (25-50 %), ++: Moderate (50-75 %), +++: Severe (75-100 %).

**Supplementary Table 5** Histopathological score showing the effect of gold nanoparticles (Au-NPs) biosynthesized by *N. oculata* algal extract against the lesions induced by lithium carbonate (Li_2_CO_3_) in brain tissue of rats.

| **Histopathological lesion** | | **C.** | **Algal Ext.** | **Au-Algal Nano-Ext.** | **Li_2_CO_3_** | **Li_2_CO_3_ treated with** | |
| --- | --- | --- | --- | --- | --- | --- | --- |
|  |  |  |  |  |  | **Algal Ext.** | **Au-Algal Nano-Ext.** |
| **Nuclear pyknosis and degeneration of the Neurons** | **Cerebral Cortex** | **-** | **-** | **-** | **+++** | **+++** | **+++** |
|  | **Hippocampus** | **-** | **-** | **-** | **+** | **-** | **-** |
| **Nuclear pyknosis and degeneration of the purkenji cell** | **Cerebellum** | **-** | **-** | **-** | **+** | **-** | **-** |

-: Nil (0-25 %), +: Mild (25-50 %), ++: Moderate (50-75 %), +++: Severe (75-100 %).
